# Intracellular trafficking and glycosylation of hydroxyproline-*O*-glycosylation module in tobacco BY-2 cells is dependent on medium composition and transcriptome analysis

**DOI:** 10.1038/s41598-023-40723-3

**Published:** 2023-08-19

**Authors:** Uddhab Karki, Paula Perez Sanchez, Sankalpa Chakraborty, Berry Dickey, Jacqueline Vargas Ulloa, Ningning Zhang, Jianfeng Xu

**Affiliations:** 1 Arkansas Biosciences Institute, Arkansas State University, Jonesboro, AR 72401 USA; 2 Molecular BioSciences Program, Arkansas State University, Jonesboro, AR 72401 USA; 3 Department of Biological Sciences, Arkansas State University, Jonesboro, AR 72401 USA; 4 College of Agriculture, Arkansas State University, Jonesboro, AR 72401 USA

**Keywords:** Molecular engineering in plants, Glycoproteins, Plant molecular biology

## Abstract

Expression of recombinant proteins in plant cells with a “designer” hydroxyproline (Hyp)-*O*-glycosylated peptide (HypGP), such as tandem repeats of a “Ser-Pro” motif, has been shown to boost the secreted protein yields. However, dramatic secretion and Hyp-*O*-glycosylation of HypGP-tagged proteins can only be achieved when the plant cells were grown in nitrogen-deficient SH medium. Only trace amounts of secreted fusion protein were detected in MS medium. This study aims to gain a deeper understanding of the possible mechanism underlying these results by examining the intracellular trafficking and Hyp-*O*-glycosylation of enhanced green fluorescent protein (EGFP) fused with a (SP)_32_ tag, consisting of 32 repeats of a "Ser-Pro" motif, in tobacco BY-2 cells. When cells were grown in MS medium, the (SP)_32_-EGFP formed protein body-like aggregate and was retained in the ER, without undergoing Hyp-*O*-glycosylation. In contrast, the fusion protein becomes fully Hyp-*O*-glycosylated, and then secreted in SH medium. Transcriptome analysis of the BY-2 cells grown in SH medium vs. MS medium revealed over 16,000 DEGs, with many upregulated DEGs associated with the microtubule-based movement, movement of subcellular component, and microtubule binding. These DEGs are presumably responsible for the enhanced ER-Golgi transport of HypGP-tagged proteins, enabling their glycosylation and secretion in SH medium.

## Introduction

Plant cell has emerged as a powerful bioproduction platform for recombinant pharmaceutical proteins since it offers advantages in *safety* and *cost*-*effectiveness* over other eukaryotic organisms^1–3^. Similar to mammalian cells, plant cells are cultured in a sterile and controlled environment and cGMP can be readily implemented throughout the production pipeline. Over the past two decades, substantial progress has been made in improving the plant cell culture system, resulting in a few commercial success cases. Notably, the FDA-approved orphan drug Elelyso^®^ is a plant cell-produced therapeutic enzyme (glucocerebrosidase) for the treatment of Gaucher’s disease, which became the world's first plant cell-made protein pharmaceutical used in humans^4,5^. Despite this breakthrough, several technical challenges limit the widespread commercial applications of this platform. The most significant challenge is low productivity, with protein yields ranging from 1.0 µg/L to 10 mg/L^6–8^. Moreover, the protein of interest tends to accumulate intracellularly, leading to increased costs of protein purification.

These technical challenges were recently addressed by a proprietary technology, termed *HypGP engineering*, which involves expressing a recombinant protein fused with a hydroxyproline (Hyp)-*O*-glycosylated peptide (HypGP) tag, which can be in the form of tandem repeats of either a “Ser-Pro” motif or an “Ala-Pro” motif, denoted (SP)_n_ and (AP)_n_ (n = 5, 10, 20, 32). The HypGP tag could function as a molecular carrier in promoting efficient transport of conjoined proteins into culture media. As a result, this technology has significantly increased the secreted yields of several recombinant proteins in plant cell cultures^9–13^ and microalgae^14^. However, it has been discovered that the secretion and Hyp-*O*-glycosylation of HypGP-tagged proteins in plant cells were greatly affected by the composition of culture medium^15^. Remarkably, fully glycosylated HypGP-tagged proteins were secreted in large quantities when BY-2 cells were grown in nitrogen-deficient Schenk and Hildebrandt (SH) medium, which resulted in low cell biomass accumulation. In contrast, small amount of secreted protein was detected when BY-2 cells were grown in nitrogen-rich Murashige and Skoog (MS) medium^16^ that supported fast cell growth and high biomass accumulation. In either case, the intracellular HypGP-tagged proteins remained non-glycosylated^9,15,17,18^. Interestingly, when the HypGP-tagged proteins were expressed in whole plants (*Nicotiana tabacum* and *Nicotiana benthamiana*), most of the synthesized proteins remained non-glycosylated either^17,19^.

This study aimed to understand the possible mechanism behind the differences in the glycosylation and secretion of HypGP-tagged proteins in BY-2 cells grown in different media. BY-2 cells expressing enhanced green fluorescence protein (EGFP) fused with a (SP)_32_ tag (32 tandem repeats of “Ser-Pro” motif)^10,15^ was mainly used as a model system to study the intracellular trafficking and Hyp-*O*-glycosylation of the recombinant protein. The reporter EGFP protein allowed us to readily conduct a subcellular localization of the synthesized proteins by confocal microscopy. BY-2 cells expressing other gene constructs, including EGFP control, (AP)_20_-EGFP (another type of HypGP tag design) and (SP)_32_-SCF (stem cell factor) were also included in this study. This was followed by the transcriptome analyses to characterize the differential gene expression of the BY-2 cells grown in SH and MS medium.

## Results

### HypGP-tagged EGFP was dramatically secreted in SH medium but not in MS medium

**Figure 1:**
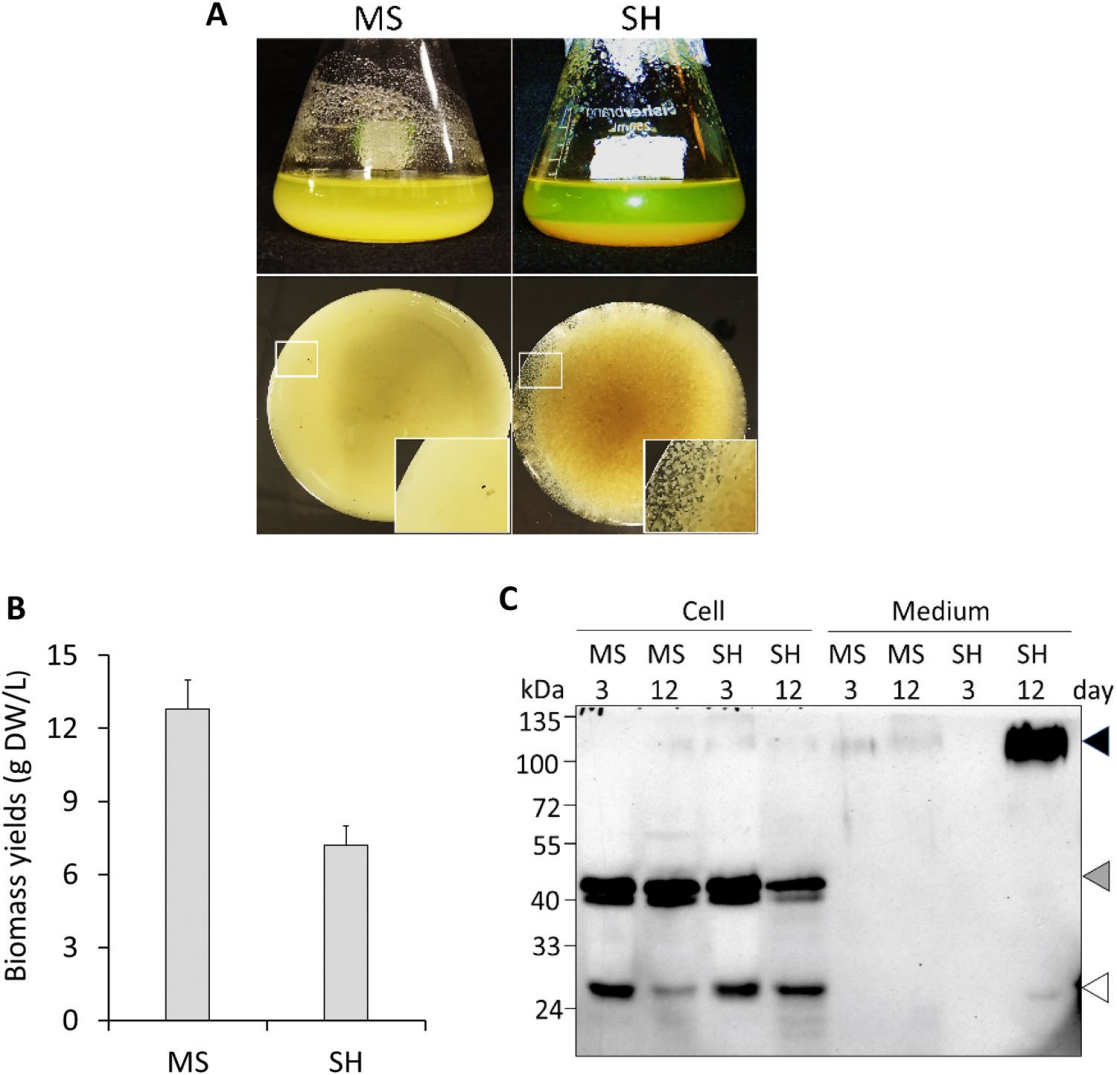
Cell growth and (SP)_32_-EGFP production of BY-2 cells cultured in MS and SH medium. **(A)** Images of BY-2 suspension culture in two different types of medium. The upper panel shows the BY-2 cell culture in flasks and the lower panel shows the plant cells viewed from the bottom of the flask. The white box shows an enlarged view of the cells; **(B)** biomass yields of the cell culture in MS and SH medium for 12 days; Error bars represent the standard deviation of three replicates (three cell cultures); **(C)** anti-EGFP Western blot detection of the (SP)_32_-EGFP secreted into media and accumulated inside cells (for original images, see Supplementary Fig. S6A). The cells and media were harvested after 3 and 12 days of culture. Proteins were extracted from the same amount of cultured cells (1.0 g fresh weight) for Western blot analysis. Fully glycosylated (SP)_32_-EGFP, non-Hyp-*O*-glycosylated (SP)_32_-EGFP and the EGFP domain cleaved from the (SP)_32_-EGFP fusion is indicated by the dark arrow, grey arrow, and white arrow, respectively.

The transgenic BY-2 cells expressing (SP)_32_-EGFP were grown in MS and SH medium, respectively for 12 days. As shown in Fig. 1, great differences in cell growth and recombinant protein production were observed between the cultures in these two types of medium. The cells grew much faster in MS medium than in SH medium, resulting in the accumulation of 1.8-fold more cell biomass in MS medium than in SH medium (Fig. 1A, B). However, the SH medium promoted extracellular secretion of the target protein, resulting in a 150.2-fold increase in the amount of secreted protein (Supplementary Table S1). Due to dramatic secretion of the (SP)_32_-EGFP protein into SH medium, the medium turned bright green (Fig. 1A). In contrast, the MS medium appeared yellowish. The green fluorescence could barely be visualized even with blue light excitation. The morphology of the BY-2 cells was also different between cultured in MS and SH medium. BY-2 cells tended to form aggregates (1 to 4 mm) in SH medium while the cells in MS medium appeared as a fine suspension culture (Fig. 1A). The observed difference in cell morphology is common for wild-type BY-2 cells and other transgenic BY-2 cell lines (cells expressing other heterologous proteins) cultured in these two types of medium (Supplementary Fig. S1).

The secretion of the fusion protein was further confirmed by the anti-EGFP Western blot analysis. While a significant amount of (SP)_32_-EGFP protein was detected in SH medium (up to 200 mg/L), a trace amount of the fusion protein was secreted into MS medium (Fig. 1C). The secreted form of the (SP)_32_-EGFP migrated at ~ 115 kDa, which was earlier identified as fully Hyp-*O*-glycosylated protein^10,15,20^. Interestingly, only non-Hyp-*O*-glycosylated forms of (SP)_32_-EGFP, migrating at 40 to 42 kDa and presumably carrying monogalactose *O*-linked to the Ser residue (42 kDa form)^17^, were present along with the cleaved EGFP domain (27 kDa) (Fig. 1C).

**Figure 2:**
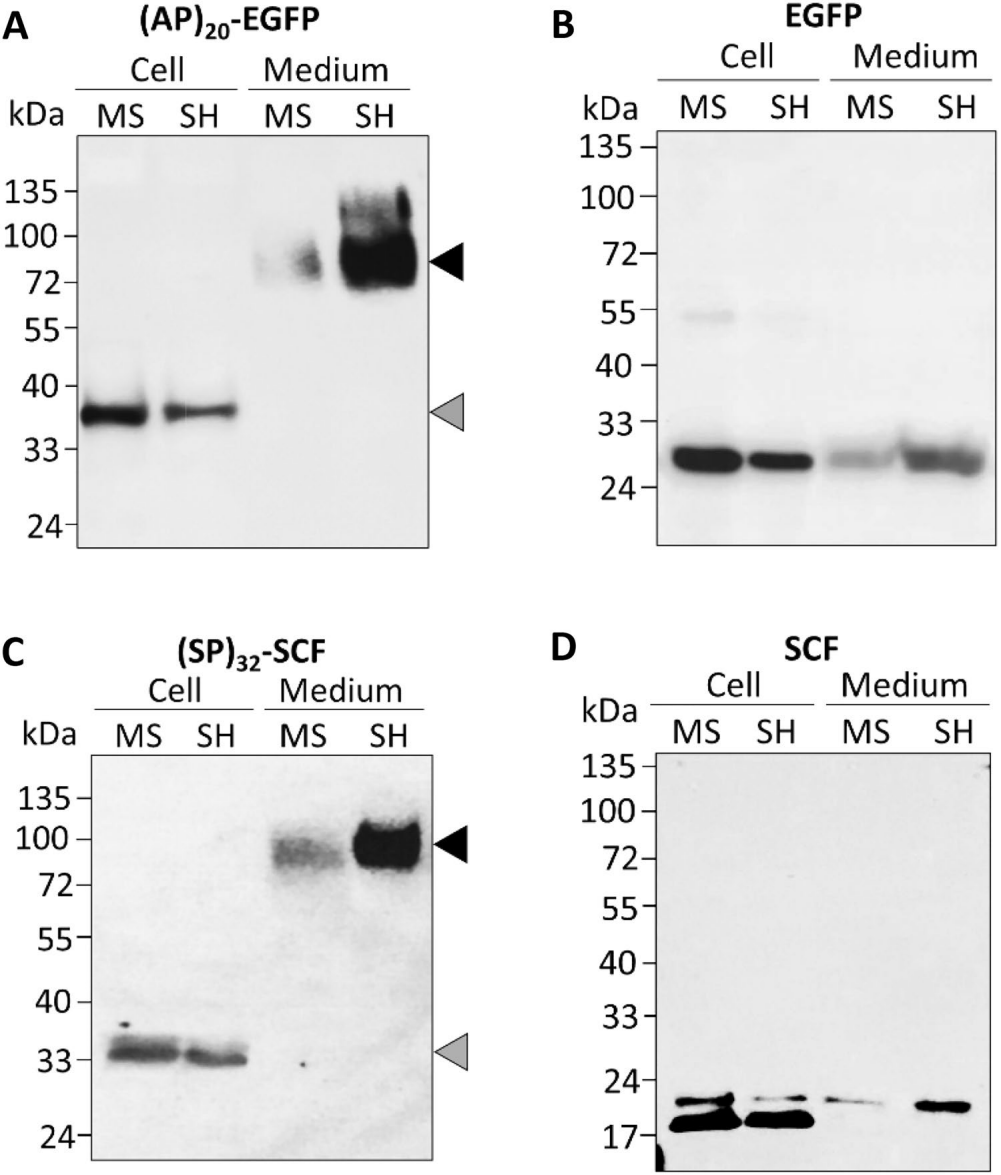
Production of different recombinant proteins by BY-2 cells grown in MS and SH medium, respectively. The cells and media were harvested after 12 days of culture. **(A,B)** Anti-EGFP Western blot detection of the (AP)_20_-EGFP and EGFP secreted into media and accumulated inside cells. **(C,D)** Anti-SCF Western blot detection of the (SP)_32_-SCF and SCF secreted into media and accumulated inside cells (for original images, see Supplementary Fig. S6B–E). In **(A,B)**, fully Hyp-*O*-glycosylated (AP)_20_-EGFP and (SP)_32_-SCF are indicated by the dark arrow, and non-Hyp-*O*-glycosylated (AP)_20_-EGFP and (SP)_32_-SCF and grey arrow, respectively. In panel **(C,D)**, upper and lower band represent the *N*-glycosylated and non-*N*-glycosylated form of SCF, respectively.

Similar result was obtained when the EGFP was expressed in BY-2 cells with a (AP)_20_ tag composed of 20 repeats of a “Ala-Pro” motif, which is a shorter HypGP chain than the (SP)_32_ tag (Fig. 2A**)**. The repetitive “Ala-Pro” motif also represents a typical Hyp-*O*-glycosylation module in plant AGPs^21^, and was reported previously to boost the secretion of fused proteins in BY-2 cell cultures^11^. In this study, while fully Hyp-*O*-glycosylated (AP)_20_-EGFP was dramatically secreted into SH medium with the secreted protein being 21.4-fold of that in MS medium (Supplementary Table S1), the non-Hyp-*O*-glycosylated form of the protein accumulated in the cells. As a control, BY-2 cells expressing EGFP only (without a HypGP tag) were also grown in SH and MS medium. Intracellular and secreted EGFP were detected in two types of medium, and both migrated into a single band of ~ 27 kDa. While intracellular EGFP in SH cells was only 54% of that in MS cells, secreted EGFP is 1.8-fold more in SH medium than in MS medium (Fig. 2B, Supplementary Table S1). The enhanced secretion of (SP)_32_ and (AP)_20_-tagged EGFP in SH medium correlated with the high degree of glycosylation of the recombinant proteins synthesized by BY-2 cells, reaching 96.2% and 92.5% for (SP)_32_-EGFP and (AP)_20_-EGFP, respectively. By contrast, only 5.3% and 31.2% of these proteins were glycosylated in MS medium (Supplementary Table S2).

### HypGP-tagged SCF was dramatically secreted in SH medium but not in MS medium

To examine if the medium effects on protein expression and secretion is protein-specific, a therapeutic protein, human stem cell factor (SCF), was also expressed in BY-2 cells with and without a N-terminal (SP)_32_ tag. Similar to the expression of (SP)_32_-EGFP or (AP)_20-_EGFP, 12.1 times more secreted (SP)_32_-SCF was detected in SH medium than in MS medium, although intracellular (SP)_32_-SCF, appearing non-Hyp-*O*-glycosylated (mw. 34 to 36 kDa), accumulated slightly more in MS cells than in SH cells (Fig. 2C, Supplementary Table S1). Again, a much higher degree of Hyp-*O*-glycosylation of the synthesized protein was detected in SH medium than in MS medium (Supplementary Table S2). As for the expression of SCF control, the secreted protein detected in SH medium were 2.2 times more than those in MS medium, though their intracellular content was similar (Fig. 2D, Supplementary Table S1), which was similar to the expression of EGFP control. In all the cases above, transgenic BY-2 cells accumulated less cell biomass in SH medium than in MS medium (Supplementary Fig. S1).

### No significant difference in the expression levels of transgene gene in BY-2 cells cultured in SH and MS medium

**Figure 3:**
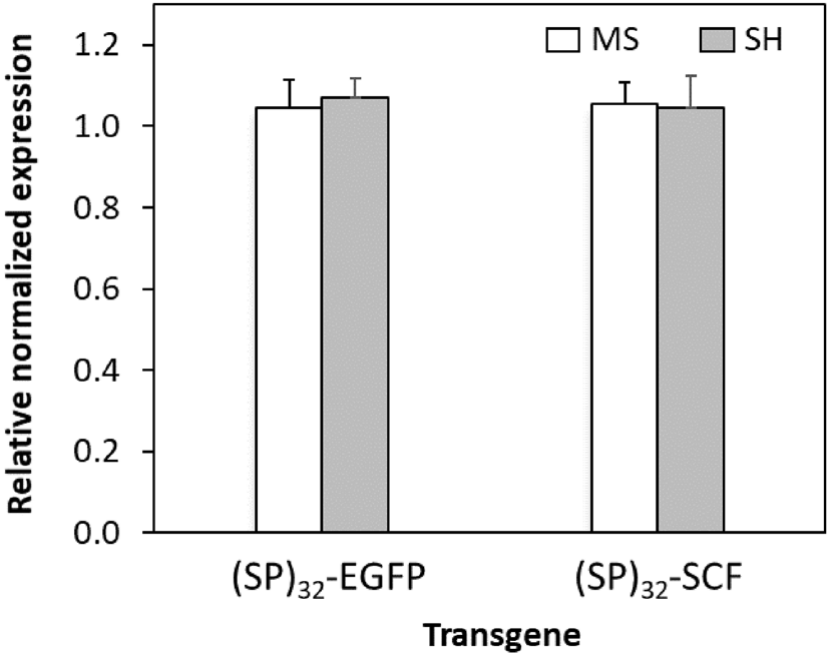
RT-qPCR detection of the expression of *(SP)*_*32*_*-EGFP* transgene in BY-2 cells grown in MS and SH medium, respectively. Three biological replicates of (SP)_32_-EGFP or (SP)_32_-SCF expressing BY-2 cells grown in SH and MS medium were collected after 8 days of subculture. There was no significant difference in the mRNA levels between the BY-2 cells cultured in SH and MS medium.

The expression level of the (*SP*)_*32*_*-EGFP* and (*SP*)_*32*_*-SCF* transgene in the BY-2 cells cultured in SH and MS medium was then determined by RT-qPCR. Interestingly, there was no significant difference in the expression levels of the two target transgenes in BY-2 cells grown at exponential growth phase (~ 8 days) (Fig. 3). Obviously, there are other factors that are responsible for the observed difference in the Hyp-*O*-glycosylation and secretion of the engineered fusion proteins between the BY-2 cells cultured in SH and MS medium.

### HypGP-tagged EGFP protein formed aggregates in BY-2 cells grown in MS medium

**Figure 4:**
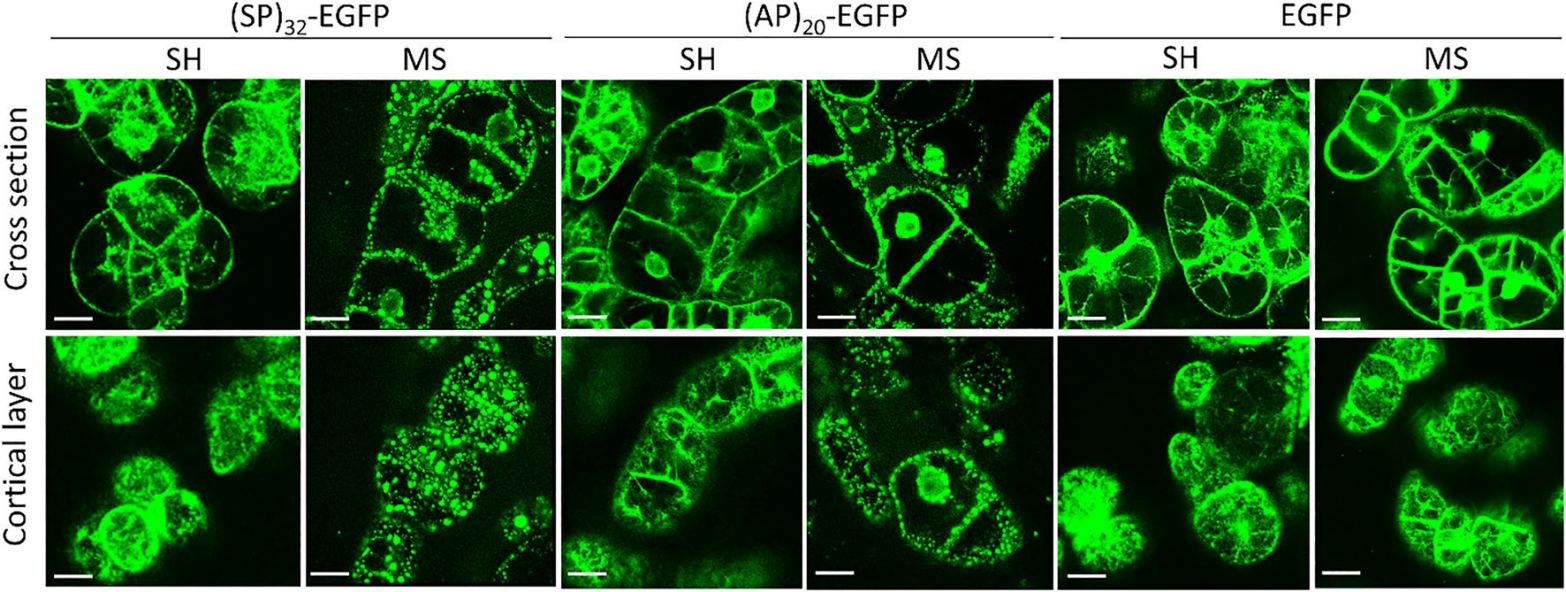
Fluorescence micrographs of transgenic BY-2 cells grown in MS and SH medium. BY-2 expressing (SP)_32_-EGFP or EGFP control were grown in both SH and MS medium for 6 days before being inspected using a laser-scanning confocal microscope. BY-2 cells were captured in both the cross section (upper panels) and the cortical cytoplasm (bottom panels) under green fluorescence channel. Scale bar = 25 µm. Fluorescent reticulate ER networks could be seen throughout the cortical cytoplasm of the BY-2 cells except the (SP)_32_-EGFP and (AP)_20_-EGFP cells grown in MS medium.

We continued to detect the subcellular localization of the synthesized (SP)_32_-EGFP under a confocal microscope. As shown in Fig. 4, when the BY-2 cells were grown in SH medium, a substantial fluorescence signal could be viewed in different organelles of cells, particularly around the nucleus and at the cell membrane, as previously reported^10^. In addition, reticulate network was observed when the cortical layer was inspected, indicating that (SP)_32_-EGFP was present in the endoplasmic reticulum (ER networks). Remarkably, many green aggregates ranging from 1 to 10 μm in size were detected when the cells grown in MS medium were inspected. These aggregates are relatively larger than the Golgi bodies, which are usually observed as punctate structures measuring 1 to 2 μm in diameter when viewed under a confocal microscope^22^. Similar results were observed in BY-2 cells expressing (AP)_20_-EGFP. Conversely, no green aggregates were observed in cells expressing the EGFP control lacking the HypGP tag. This finding suggests that the (SP)_32_ or (AP)_20_ module was potentially responsible for the formation of these green aggregates.

**Figure 5:**
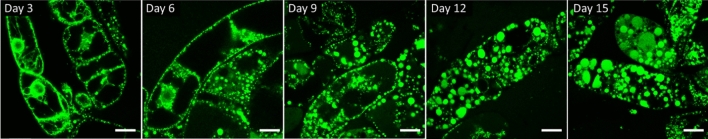
Fluorescence micrographs of BY-2 cells expressing (SP)_32_-EGFP after growing in MS medium for different time. BY-2 cell images were captured in cross section under green fluorescence channel using a laser-scanning confocal microscope. Scale bar = 25 µm.

The formation of the green aggregates was also dependent on the culture time (Fig. 5). At the lag growth phase (Day 3), when the synthesis of recombinant protein just started, the intracellular distribution of the green fluorescence was similar to that of EGFP control cells, although some small aggerates (~ 1 μm in diameter) could be observed in some cells. While the cells grew rapidly from day 6 to day 12, much more green fluorescent aggregates were formed, and the size of the aggregates increased up to 10 μm. At the end of the culture, aggregates with a diameter of 13 μm could be found. This indicated that the accumulation of the green fluorescent aggregates was a dynamic process, and it was related to the cell growth stage (protein synthesis rate).

### (SP)_32_-EGFP aggregates are retained in ER

**Figure 6:**
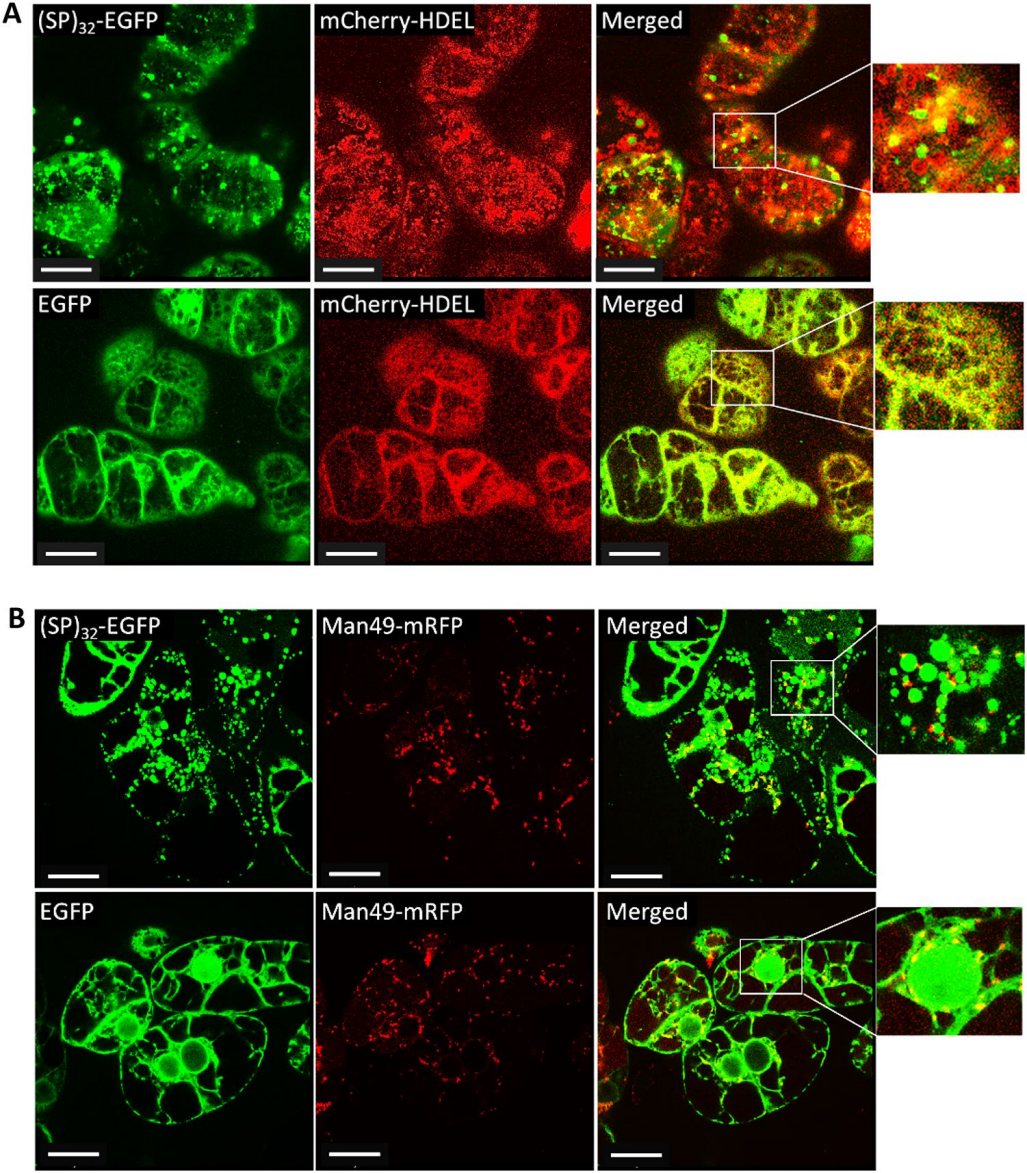
Subcellular localization of (SP)_32_-EGFP and EGFP expressed in BY-2 cells. **(A)** colocalization of (SP)_32_-EGFP or EGFP control with ER marker (mCherry-HDEL); (**B**) colocalization of (SP)_32_-EGFP or EGFP control with Golgi marker (Man49-mCherry). Transgenic BY-2 cells were grown in MS medium for 6 days before being inspected using a laser-scanning confocal microscope. Cell images were captured under both green fluorescence channel and red fluorescence channel with a 100× oil-immersion objective. The white box shows an enlarged view of the image; Scale bar = 25 µm.

To investigate the subcellular localization of the (SP)_32_-EGFP aggregates in plant cells, BY-2 cells expressing (SP)_32_-EGFP were further transferred with gene encoding an ER marker (mCherry-HDEL) and a Golgi marker (Man49-mCherry), respectively. The results, shown in Fig. 6A, indicated that almost all of the green fluorescent aggregates were located in the ER network area; in other words, they overlapped with the red fluorescent ER marker. This was the same as the expressed EGFP control, which is always detected to colocalize with the ER network as it is transported through the default ER-Golgi pathway for secretion. When (SP)_32_-EGFP cells were co-transformed with the Golgi marker, the green fluorescent aggregates were segregated with the red discrete Golgi bodies, while most of the expressed EGFP control overlapped with this organelle during transport through the ER-Golgi pathway **(**Fig. 6B**)**. This indicated that the synthesized (SP)_32_-EGFP fusion protein could be retained in the ER without exiting and being subsequently transported to the Golgi apparatus. This collaborated with the observation that virtually all the intracellular fusion proteins were not Hyp-*O*-glycosylated (Fig. 1C), as the Hyp-*O*-glycosylation occurred in the Golgi apparatus^23^.

### BFA treatment induced the formation of (SP)_32_-EGFP aggregates

**Figure 7:**
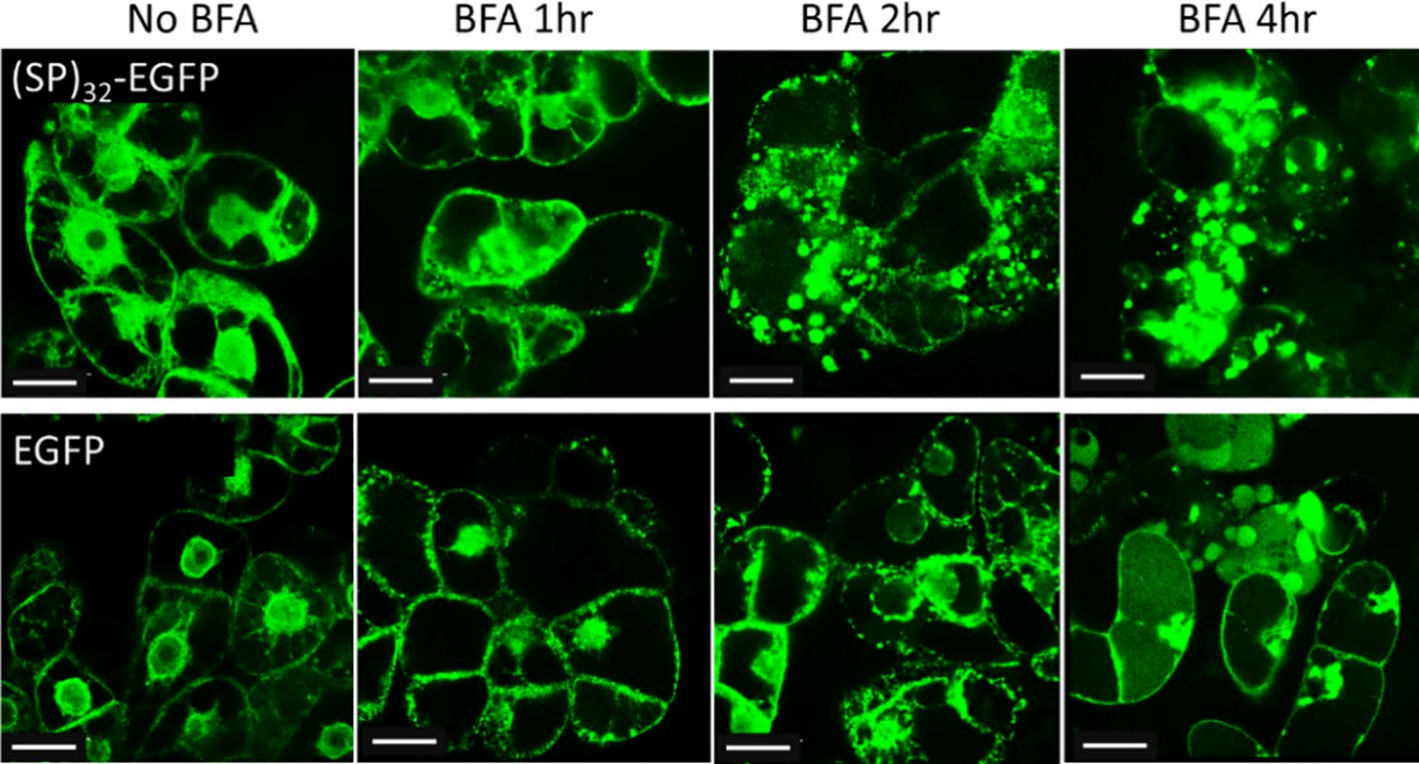
Fluorescence micrographs of transgenic BY-2 cells treated with BFA. The BY-2 cells expressing (SP)_32_-EGFP or EGFP were grown in SH medium for 6 days, and then treated with BFA for 1, 2 and 4 h. The cells were captured under green fluorescence channel with a 100× oil-immersion objective. Scale bar = 25 µm. Green fluorescent aggregates could be seen after 1 h treatment for both types of cells. More and larger aggregates formed with prolonged BFA treatment up to 4 h.

In order to further understand the mechanism responsible for the formation of green fluorescent aggregates in (SP)_32_- or (AP)_20_-taggged EGFP cells and the subcellular localization of the aggregates, BY-2 cells expressing (SP)_32_-EGFP were treated with BFA, a fungal toxin that blocks protein transport between the ER and the Golgi apparatus by quickly destroying the Golgi stacks^24^. As a control, BY-2 cells expressing EGFP control were also treated in the same way. As shown in Fig. 7, BFA treatment significantly changed the subcellular localization of the (SP)_32_-EGFP. One hour after the incubation with BFA, small green fluorescent aggregates of 1–2 μm could be observed in the cells. With prolonged incubation with BFA to 2 and 4 h, a much greater number of green fluorescent aggregates formed and the diameter of the aggregates also increased up to 8 μm. This was the same as the early report in which protein aggregates formed following the BFA treatment in hairy roots expressing (SP)_32_-EGFP^17^. Interestingly, BFA treatment also induced the formation of green fluorescent aggregates in EGFP-expressing cells, and the process was the same as that in the (SP)_32_-EGFP cells. These changes indicated that BFA treatment successfully blocked the transport of synthesized (SP)_32_-EGFP and EGFP from ER to Golgi apparatus, which caused the accumulation of the (SP)_32_-EGFP or EGFP in ER and formation of green fluorescent aggregates.

### RNA sequencing and sequences mapping to reference genome

To reveal a comprehensive molecular mechanism underlying higher secretion of HypGP-tagged protein in BY-2 cells cultured in SH media, but formation of protein aggregates/protein bodies in MS medium, total mRNA was sequenced to identify differential gene expression in the BY-2 cells cultured in SH media with MS medium (as a control). Here, the wild-type BY-2 cell instead of transgenic BY-2 cell was used for transcriptome analysis in order to avoid the influence of genetic transformation and continuous elite cell lines selection on the characteristic of the cell line, because these processes potentially led to some additional mutations of the cell line^25^. In addition, the observed results, including enhanced Hyp-*O*-glycosylation and protein secretion, were common to the expression of different HypGP-tagged proteins in BY-2 cells, indicating that the medium effects were independent of the heterologous genes integrated into the genome of the cells.

Three RNA-seq libraries were constructed for BY-2 cells cultured in MS and SH medium, respectively (three biological replicates for each culture medium). The raw reads generated from each library ranged from 20,250,845 to 22,832,945. After removing the low-quality reads and adapter sequences, the total clean reads were mapped with the genome of *N. tabacum*. The results showed that 95.81–96.76% of total reads of the six libraries were unambiguously mapped to *N. tabacum* genome, of which more than 93% were mapped to unique locations. The sequencing data and mapping of RNA-seq data were summarized in Supplementary Table S4. The gene expression level was quantified by the abundance of transcripts that mapped to exons. The Pearson correlation coefficient between the samples and distribution of gene expression under the two culture conditions is shown in Supplementary Fig. S2.

### Identification of differentially expressed genes (DEGs)

**Figure 8:**
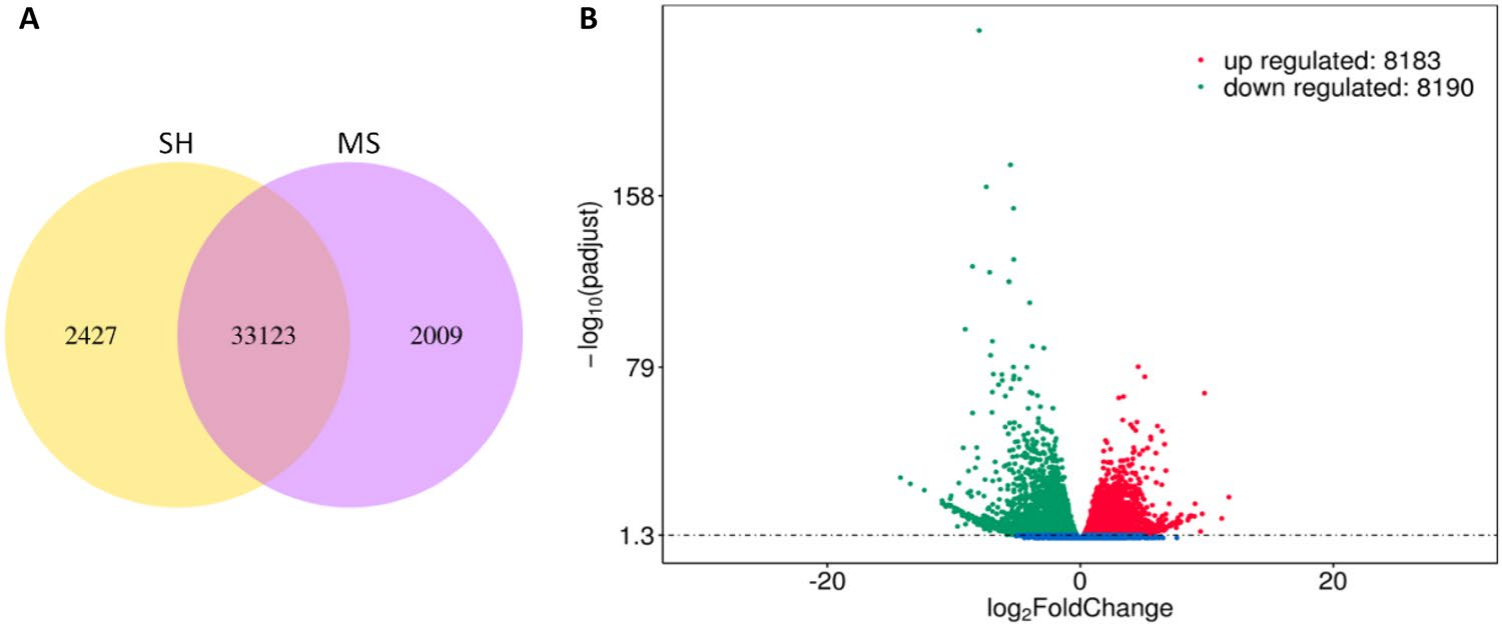
Expression genes in BY-2 cells grown in SH medium as compared with those in MS medium. **(A)** Venn diagram of expression genes; **(B)** volcano plot of DEGs. The abscissa shows the fold change difference in the expression of genes in different groups, and the vertical coordinates indicate the *p*-values for the differences in expression. The upregulated genes are represented by red dots, and the downregulated genes are represented by green dots. Genes without significant differences are indicated by blue dots.

DEGs were identified by comparing gene expression of BY-2 cells cultured in SH with that in MS medium using DESeq2 with a *p*-value < 0.05^26^. It was found that 16,373 genes were differentially expressed, including 8183 upregulated and 8190 downregulated genes (Fig. 8). The list of highly downregulated genes was dominated by genes encoding cell wall structural proteins, such as vegetative cell wall protein gp1-like, proline rich and glycine rich cell wall structural proteins, as well as cell wall modifying enzymes and proteases, such as probable polygalacturonase and aspartyl protease AED3-like, although genes encoding osmotin-like protein, zinc transporter 11-like and purple acid phosphatase as well as many uncharacterized genes were notable (Supplementary Table S5). In contrast, genes encoding alkane hydroxylase MAH1-like, auxin-responsive protein IAA9-like, phospholipase A1, senescence-specific cysteine protease-like, tubulin beta-1 chain, and many uncharacterized genes were found to be upregulated (Supplementary Table S6).

Although not highly up- or down- regulated, genes encoding prolyl 4-hydroxylase (P4H) catalyzing the hydroxylation of proline residue and two key groups of glycosyltransferases responsible for building the Hyp-*O*-glycans, Hyp-*O*-galactosyltransferase (Hyp:GlaT) and β-1,3-galactosyltransferase (Gal:GalT), was also analyzed for their expression patterns. Five DEGs for P4H, 3 DEGs for Hyp:Gla, and 5 DEGs for Gal:GalT were identified. While most of the genes encoding P4H and Hyp:GlaT isoforms were down-regulated when the BY-2 cells were grown in SH medium, genes encoding all the four Gal:GalT isoform genes were upregulated in SH medium (Supplementary Fig. S3).

### Functional enrichment analysis of the DEGs

**Figure 9:**
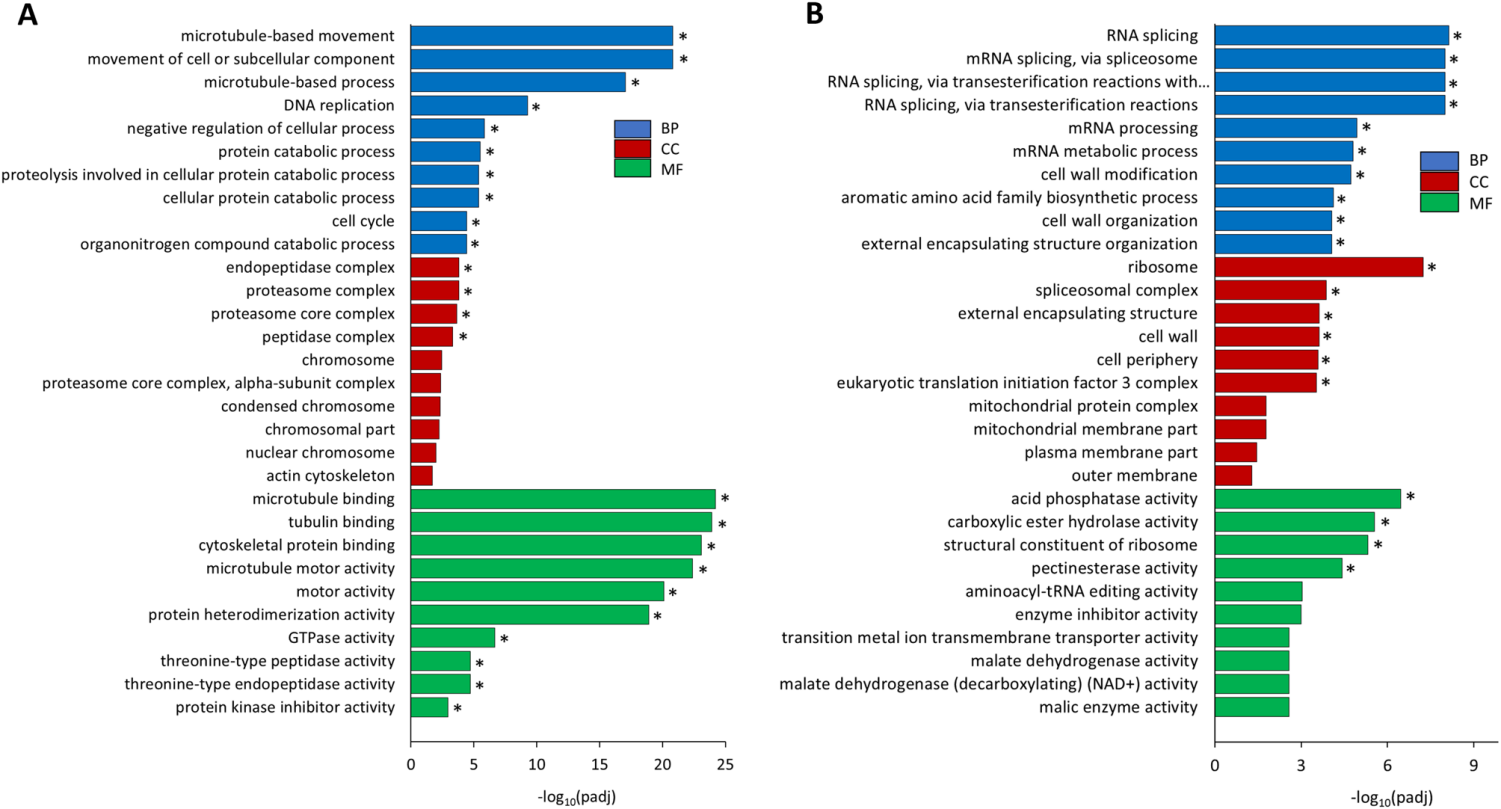
GO enrichment analysis of BY-2 cells cultured in SH medium as compared to MS medium. Upregulated GO terms (**A**) and downregulated GO terms **(B)** specific to the biological process (BP), cellular components (CC), and molecular function (MF) are presented. Top 10 GO terms under each category are ranked in descending order by adjusted *p*-value [− log_10_(padj)]. Asterisk indicates significant difference at *p* < 0.05.

To get functional annotation of the DEGs datasets, we aligned all the DEGs against the GO and KEGG databases. Based on our results, 68 GO terms were significantly enriched by upregulated genes, and 23 GO terms were enriched by downregulated genes. Some noteworthy *biological processes*, including movement of cell or subcellular component, microtubule-based movement, negative regulation of cellular process, regulation of cellular component organization, protein catabolic process, and cell cycle, were associated with overexpressed genes in BY-2 cells cultured in SH medium. In addition, upregulated genes enriched four *cellular components,* including proteasome complex, endopeptidase complex, proteasome core complex and peptidase complex, and over ten *molecular functions* including but not limited to microtubule binding, tubulin binding, protein heterodimerization activity and protein kinase inhibitor activity, etc. (Fig. 9A). On the other hand, RNA splicing, cell wall modification, cell wall organization/biogenesis, protein import were some of the remarkable *biological processes* enriched by downregulated genes. Notably, we found that cell wall, ribosome, and cell periphery were some of the remarkable *cellular constituents* enriched by downregulated genes. Also, four *molecular functions*, including acid phosphatase activity, carboxylic ester hydrolase activity, pectinesterase activity, and structural constituent of ribosome, were overrepresented by downregulated genes (Fig. 9B). From the analysis of KEGG pathway, we found that DNA replication and phagosome pathway had significant enrichments by upregulated genes and spliceosome pathway by downregulated genes (Supplementary Fig. S4).

### RT-qPCR validated notable DEGs in BY-2 cells cultured in SH medium vs. MS medium

To further confirm the differential regulation of genes identified in RNA-Seq analysis, we cross-validated two upregulated genes, *alkane hydroxylase MAH1-like gene* (LOC107798865) and *laccase-15-like gene* (LOC107829253), and two downregulated genes, *polygalacturonase gene* (LOC107824442) and *vegetative cell wall protein gp1-like gene* (LOC107802007) by RT-qPCR. Our data confirmed that the *alkane hydroxylase MAH1-like gene* and *laccase-15-like gene* were significantly upregulated by 6.0-folds and 3.2-folds, respectively in the BY-2 cells cultured in SH medium (Supplementary Fig. S5). The former one is an integral component of membrane having molecular function of oxidoreductase activity and iron ion binding^27^, and the latter is involved in lignin synthesis in plants^28^. Similarly, the two highly downregulated genes, including that encoding polygalacturonase and that encoding vegetative cell wall protein gp1-like, showed consistent results in RT-qPCR analysis (Supplementary Fig. S5). Polygalacturonase is a secretory enzyme localized in cell wall of plant cells, and its functions is to randomly hydrolyze (1–4)-α-d-galactosiduronic linkages in pectate and other galacturonans^29^. The vegetative cell wall protein gp1 is a hydroxyproline-rich glycoprotein (HRGP)-1 that functions as a major component of the outer cell wall (crystalline) layer^30^.

## Discussion

Expression of heterologous proteins with a HypGP tag, such as (SP)_n_ and (AP)_n_, in plant cells dramatically increased the secreted yields of the proteins. This has been demonstrated in the expression of many proteins in plant cells (tobacco and Arabidopsis)^9–13,18^ as well as in microalgae^14^. In terms of functionality, the extensively Hyp-*O*-glycosylated HypGP tag has been found to greatly extend the serum half-life of small therapeutic proteins, such as human growth hormone and interferon α2, without significantly affecting their bioactivity^12,13^. In addition, the HypGP tag decorated with Hyp-glycans were found not immunogenic when injected into mice and only mildly so when injected as a fusion protein^12^. However, for those therapeutic proteins whose clinical applications require them to be in a native form, a specific proteolytic cleavage site, for example that is recognized by enterokinase, could be designed between the HypGP tag and the target proteins.

Interestingly, the secretion and Hyp-*O*-glycosylation of HypGP-tagged proteins in BY-2 cells are highly dependent on the culture medium. While high secreted protein yield was obtained in SH medium, trace amount of secreted protein was detected in MS medium^15,17^. Furthermore, in both types of medium, the expressed proteins appeared as different glycoforms and were completely segregated between the medium fractions (fully glycosylated form) and the cells (non-Hyp-*O*-glycosylated form)^10,17^. This study strived to understand the possible molecular mechanism behind the observed difference in the glycosylation and secretion of HypGP-tagged proteins by studying the intracellular trafficking of the synthesized proteins and analyzing the transcriptome of the BY-2 cells grown in SH and MS medium. Considering that the designer (SP)_n_ and (AP)_n_ tag are the signature glycosylation modules of AGPs, one of major plant cell wall glycoproteins^31,32^, the dissection of the intracellular trafficking and Hyp-*O*-glycosylation of these modules will also advance our understanding of the biosynthesis and functions of plant cell wall structural glycoproteins.

It was found that the medium effects on the glycosylation and secretion of synthesized protein is common to the expression of different HypGP-tagged proteins, including different designs of HypGP tag [(SP)_32_ and (AP)_20_] and different types of proteins (EGFP and SCF) (Figs. 1, 2). However, the effect was not as obvious when the non-HypGP-tagged protein (EGFP and SCF) was expressed in BY-2 cells **(**Fig. 2B, D), though the plant cells always grew slower in SH medium than in MS medium (Supplementary Fig. S1). RT-qPCR revealed that there was no significant difference in the transcription levels of the transgenes (*SP*)_*32*_*-EGFP* and (*SP*)_*32*_*-SCF* in BY-2 cells cultured in these two types of media (Fig. 3). This also indicated that the constitutive 35SCaMV promoter that drives the expression of the transgenes in BY-2 cells was independent of the culture medium. Given that an identical mRNA sequence was produced in comparable amounts in BY-2 cells cultured in two different media, it can be inferred that the resulting protein synthesis through translation is also comparable. Therefore, other posttranslational factors would be responsible for the observed difference in the Hyp-*O*-glycosylation and secretion of the engineered HypGP module.

Further examining the subcellular localization of synthesized (SP)_32_- and (AP)_20_-tagged EGFP cells found that many green fluorescence aggregates formed in the ER of BY-2 cells grown in MS medium (Fig. 4), and their size increased with time (Fig. 5). This corroborated with the observations that HypGP-tagged proteins were barely secreted into MS medium, and the intracellular fusion proteins were not Hyp-*O*-glycosylated, because Hyp-*O*-glycosylation occurs in Golgi. By contrast, such aggregates were absent from the BY-2 cells grown in SH medium, which led to dramatic secretion of the fully Hyp-*O*-glycosylated proteins. Interestingly, no green fluorescent aggregates were observed in the BY-2 cells expressing EGFP control, either cultured in MS or SH medium (Fig. 4). Obviously, the HypGP tag was responsible for the formation of the green fluorescent aggregates in the BY-2 cells grown in MS medium, so the synthesized protein retained inside plant cells.

The observed EGFP aggregates were like the protein bodies (PBs) which occur naturally as storage organelles in seeds^33^. PBs were previously reported to form in the ER of plant leaves, such as in* N. benthamiana* and *N. tabacum*, when foreign proteins were synthesized at high levels in the ER and when fused to one of three special tags: Zera (a domain of the maize seed storage protein gamma-zein), elastin-like polypeptides (ELP), or hydrophobin-I (HFBI)^34–38^. Formation of PBs in plant cells, such as BY-2 cells, could also be induced by fusion with HFBI^25,39^. In the case of expression of HypGP-tagged proteins in BY-2 cells, the synthetic HypGP module, such as (SP)_32_ or (AP)_20_ seemed to act like the ELP or HFBI tag to induce the formation of the protein aggregates in ER. However, this occurred only when the BY-2 cells were grown in MS medium, not in SH medium, which was different from the function of ELP and HFBI tags that always included the formation of PBs in ER. Our study has also demonstrated that BFA treatment of the BY-2 cells, which retained the secretory proteins in ER, induced the formation of green fluorescent aggregates of (SP)_32_-EGFP in SH medium or even the EGFP control (Fig. 7). Formation of PBs in plant cells solely due to high-level accumulation of recombinant proteins in ER (> 0.2% of total soluble protein) without the need for a fusion tag was previously reported^34^. In our case, the presence of the synthetic HypGP tag might simply prevent the ER exit of the fusion protein when the BY-2 cells grow in MS medium, thus inducing the formation of PBs-like aggregates. To this end, substantial variations in the biological and cellular processes must occur between the plant cells grown in these two types of medium.

As reported earlier, the major difference between the SH and MS medium is the *macronutrients*, specifically the nitrogen content^15^. The MS medium contains not only a significantly higher total nitrogen content compared to the SH medium (60.1 mM in MS vs. 27.3 mM in SH), but also a substantial concentration of NH_4_^+^ (Supplementary Table S3). As a result, the NO_3_^−^/NH_4_^+^ ratio in MS medium is much lower than that in SH medium, with a ratio of 1.9 in MS and 9.5 in SH. The observed low cell biomass accumulation in SH medium was likely caused by insufficient supply of nitrogen in the “nitrogen-starvation” SH medium and/or unbalanced NO_3_^-^/NH_4_^+^ ratio. Interestingly, modifying the MS medium macronutrients with reduced NH_4_NO_3_ contents (from 1/2 of its original level to NH_4_NO_3_ free) could trigger high-yield secretion of the fully glycosylated (SP)_32_-EGFP, although the cell biomass was greatly reduced^15^. Nitrogen source and availability have been regarded as critical factors for the productivity of plant cell cultures as it plays a pivotal role in plant cell metabolism and is directly connected to amino acid and protein biosynthesis^40^. Supplementing plant cell cultures with additional nitrogen was previously reported to enhance the accumulation of recombinant proteins^41,42^. However, when HypGP-tagged proteins were expressed in plant cells, the use of medium with high nitrogen levels, such as MS medium, caused the fusion protein to be retained in the ER and form PBs-like aggregates, although it promoted cell growth. Only a medium with lower nitrogen levels, such as SH medium, was found to be more suitable for promoting the ER exit of fusion proteins, allowing for subsequent Hyp-*O*-glycosylation and secretion.

In addition to nitrogen, one of the major *micronutrients*, Zn^2+^ (ZnSO_4_) is also substantially lower in concentration in SH medium than in MS medium (8.6-fold less). However, the concentration of this ion did not show any effect on the Hyp-*O*-glycosylation and secretion of the HypGP-tagged proteins in BY-2 cells^15^, although the gene encoding zinc transporter 11-like protein was found to be significantly downregulated in the SH medium (Supplementary Table S5). The same is true for Mn^2+^ and Fe^2+^ ions; their concentrations in SH medium are about half of those in MS medium (Supplementary Table S3), but they did not impact the secretion of HypGP-tagged proteins^15^.

The striking difference in cell growth, cell morphology and secretion of recombinant proteins observed between BY-2 cells grown in MS and SH medium, especially in terms of intracellular transport and Hyp-*O*-glycosylation of the HypGP-tagged proteins, suggests significant changes in biological processes occurred within the cells. This prompted us to conduct RNAseq analysis to understand the transcriptomic response of BY-2 cells to nitrogen-deficient SH media. The mapping of reads with the genome of *N. tabacum* was 95–96% (Supplementary Table S4), which was due to slight mutation of the BY-2 cell line that has been continuously cultured in vitro since 1981^43^. It is worth noting that over 16,000 genes were found to be differentially expressed, even though both SH and MS medium could properly support the propagation of BY-2 cells in vitro. Transcriptome-wide analysis was reported earlier on BY-2 cells in response to treatment with methyl jasmonate. The results also revealed a large number of differentially expressed transcripts, with a total of 7260 transcripts, showing significant changes in expression levels^44^. These findings suggest that the in vitro cultured BY-2 cells are highly sensitive to changes in their environment. They can mount a significant response to external stimuli such as medium composition and MeJA treatment.

The observed DEGs, including those down- and up- regulated in the transcriptome data (Supplementary Table S5, S6), clearly indicate that nitrogen-deficient SH media had great impact on a variety of cellular structures, cellular processes, and metabolic pathways. Obviously, BY-2 cells cultured in SH medium induced stress compared with MS medium, resulting in significant downregulation of the expression of cell wall structural proteins (e.g. vegetative cell wall protein gp1, proline-rich and glycine-rich protein). The nitrogen deficiency also caused the reduced expression of osmotin-like protein that plays an important role in plant immune systems during stress conditions, as well as several proteases and cell wall modifying enzymes. It is worth noting that a pectin hydrolase, polygalacturonase, was downregulated to the greatest extent in SH medium (Supplementary Table S5). Pectin is considered as “glue” between two cells in plant cells where pectin-rich middle lamella mediates cell–cell adhesion^45^. Thus, downregulation of the pectin hydrolyzing enzyme polygalacturonase might be responsible for the observed clumping of BY-2 cells cultured in SH medium. On the other hand, the substantial upregulation of the expression of alkane hydroxylase MAH1-like, dirigent protein 23-like, and superoxide dismutase, etc. also suggests that the plant cells are responding to some form of stress, and that they are attempting to adapt to its environment in order to survive and thrive. Our results are consistent with some other studies examining the effects of nitrogen deficiency on various biological processes in plants. For instance, Rivai et al. (2021) found that sorghum seedlings grown in nitrogen-deficient media exhibited significant changes in cell wall and its modifying genes based on RNAseq and RT-qPCR data^46^. Also, nitrogen deficiency in cucumber seedlings was found to affect cell wall remodeling^47^, and deficiency in maize root in its developmental stage strongly altered various biological regulations, including protein modification, protein degradation, signal transduction, and hormonal regulation^48^.

Considering that hydroxylase and glycosyltransferases play an important role in the Hyp-*O*-glycosylation and subsequent secretion of HypGP-tagged recombinant proteins, the expression profiles of the genes encoding these enzymes were examined (Supplementary Fig. S3). While the genes encoding the P4H and Hyp:GlaT isoforms did not show consistent expression regulation in SH medium, all the five Gal:GalT isoform genes were substantially upregulated. This finding is consistent with the two-stage Hyp-*O*-glycosylation model proposed earlier. In this model, hydroxylation of Pro and the addition of the first galactose to Hyp residue in ER, which are catalyzed by P4H and Hyp:GlaT enzymes, respectively, may be an independent and highly efficient event. However, the subsequent extension of the galactose backbone involving the addition of more galactose residues to the Hyp-linked galactose, triggered by Gal:GalT enzyme, comprises a rate-limiting step^49–51^.

GO analysis helped to highlight the biological processes mostly involved in the response to the nitrogen-deficiency SH medium. As seen in Fig. 9A, the upregulated genes were significantly overrepresented in the cellular response to microtubules-based movement and process, movement of cell and cellular component, stress-based proteasome complex, microtubules and tubulin binding, and microtubule motor activity. Microtubules are an essential component of the plant cell cytoskeleton, which is involved in various cellular processes such as cell division, cell elongation, and organelle movement^52^. The upregulation of genes involved in microtubule-based movement and microtubule motor activity suggests that the BY-2 cells responded to the SH medium environment by altering their cytoskeleton to compensate for the nutrient deficiency. Although microtubules are not necessary for ER-to-Golgi protein transport, they can facilitate the movement of transport carriers from the ER to the Golgi complex when present^53^. Therefore, the significantly upregulated genes associated with the microtubules-based movement and microtubules and tubulin binding presumably contributed to the enhanced ER-to-Golgi transport of HypGP-tagged proteins and other proteins in BY-2 cells grown in SH medium, which led to Hyp-*O*-glycosylation of proteins in Golgi and subsequent extracellular secretion. GO and KEGG analysis also demonstrated the substantial downregulation of the genes associated with protein synthesis in BY-2 cells cultured in SH medium, including RNA splicing, mRNA processing, aromatic amino acid biosynthesis, ribosome, spliceosomal complex, translation initiation factor 3 complex etc., and the activities of several enzymes, such as acid phosphatase and carboxylic ester hydrolase (Fig. 9B**)**, which was presumably responsible for the observed reduced plant cell growth in SH medium.

Due to the large number of DEGs detected in BY-2 cells grown in SH medium as compared with MS medium, the transcriptome analysis could not yield a direct explanation for the observed effect of the media on the intracellular trafficking and Hyp-*O*-glycosylation of HypGP-tagged proteins. However, the transcriptome data do provide valuable insights into the cellular response of BY-2 cells to changes in the medium composition. These findings underscore the sensitivity of BY-2 cells to their culture environment and suggest that the medium composition can have a significant impact on cell growth and recombinant protein production/secretion. Future studies could focus on investigating specific genes and pathways involved in these processes to gain a better understanding of the molecular mechanisms underlying plant cells’ responses to nitrogen deficiency.

## Conclusion

The intracellular trafficking and Hyp-*O*-glycosylation of HypGP-tagged proteins (EGFP and SCF) in BY-2 cells were strongly influenced by the culture medium. The presence of synthetic HypGP tags, such as (SP)_32_ and (AP)_20_, was found to hinder the fusion protein's exit from the ER in MS medium, leading to the formation of PBs-like aggregates. In contrast, SH medium facilitated efficient transport of HypGP-tagged proteins to the Golgi, enabling Hyp-*O*-glycosylation and subsequent dramatic secretion. Transcriptome analysis revealed a large number of DEGs (over 16,000) in BY-2 cells grown in SH medium compared to the MS medium control, with many upregulated DEGs associated with microtubule-based movement, movement of cells or subcellular components, and microtubule binding. These DEGs were presumably responsible for the enhanced ER-Golgi transport of HypGP-tagged proteins for subsequent glycosylation and secretion. The findings of this study highlight the need for further research into the impact of the medium composition on recombinant protein production/secretion in plant cell cultures, which could ultimately lead to the development of more efficient and cost-effective protein expression systems. Furthermore, this study also improves our understanding of the intracellular trafficking and Hyp-*O*-glycosylation process of plant cell well glycoproteins in plant cells, as the HypGP tags, including (SP)_32_ and (AP)_20_, are the signature modules of arabinogalactan glycoproteins in the plant cell wall.

## Methods

### Expression vectors and BY-2 cell transformation

**Figure 10:**
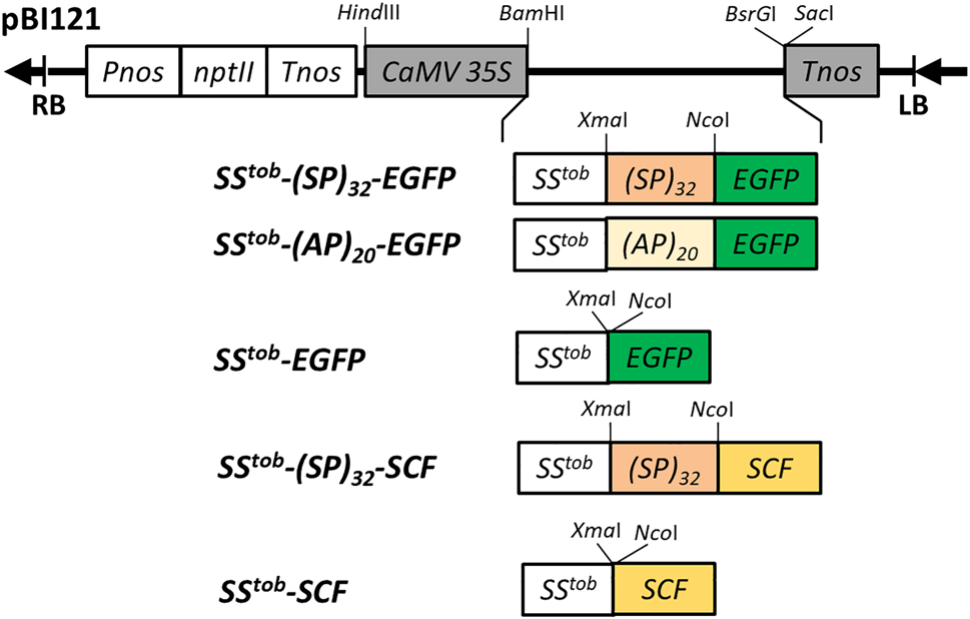
Schematic of the gene constructs cloned in *pBI121* expression vector. *SS*^*tob*^ tobacco extensin signal sequence; *(SP)*_*32*_ 32 tandem repeats of a “Ser-Pro” motif; *(AP)*_*20*_ 20 tandem repeats of a “Ala-Pro” motif; *SCF* human stem cell factor; *CaMV35S* 35S cauliflower mosaic virus promoter; *Pnos* nopaline synthase promoter; *Tnos* nopaline synthase terminator; *nptII* neomycin phosphotransferase II.

Transformed BY-2 cells expressing EGFP control, (SP)_32_-EGFP and (AP)_20_-EGFP, all proceeded by the *35S cauliflower mosaic virus* promoter (*CaMV*35S) promoter and a tobacco extensin signal peptide sequence (SS^tob^) (Fig. 10) were created earlier^9,15^. *Human stem cell factor (SCF)* gene was amplified by PCR from the *pBI121-SCF-(SP)*_*20*_ template^9^, and subcloned into *pBI121-SS*^*tob*^*-(SP)*_*32*_*-EGFP*^20^ at the *Xma*I and *BsrG*I sites to generate *pBI121-SS*^*tob*^*-(SP)*_*32*_*-SCF* (Fig. 10). The vector was transferred into *Agrobacterium tumefaciens* LBA4404 by the freeze–thaw method, and then stably transformed into BY-2 cell by using the *Agrobacterium*-mediated method^54^. The tobacco BY-2 cells were obtained from the lab of Dr. Marcia Kieliszewski at Ohio University (Athens, OH, USA). Transformed BY-2 cell colonies were selected by 100 µg/ml kanamycin. Golgi marker encoded by *pFGC-Man49-mCherry* and ER marker encoded by *pFGC-mCherry-HDEL*^55^ were obtained from the Arabidopsis Information Resource (TAIR) (Columbus, OH). Each of them was stably transformed into the BY-2 cell line expressing (SP)_32_-EGFP or EGFP control for subcellular colocalization analysis. Transformed cell colonies were selected by 5 µg/ml bialaphos. All the research involving recombinant DNA and transgenic plant cells complied with the NIH Guidelines for Research Involving Recombinant or Synthetic Nucleic Acid Molecules, and followed the protocol that was approved by the Institutional Biosafety Committees (IBCs) of Arkansas State University (Approval #: 95565-4).

### Transgenic BY-2 cell culture and cell biomass determination

Transformed BY-2 cells were maintained in either SH medium consisting of 3.2 g/L SH basal salt mixture (PhytoTech Labs, St. Lenexa, KS), 2.1 mg/L *p*-chlorophenoxyacetic acid, 0.4 mg/L 2,4-dichlorophenoxyacetic acid (2,4-d), 0.1 mg/L kinetin, and 34 g/L sucrose, or MS medium consisting of 4.3 g/L MS basal salt mixture (PhytoTech Labs), 0.18 g/L KH_2_PO_4_, 100 mg/L myo-inositol, 1 mg/L thiamine, 0.44 mg/L 2,4-d, and 30 g/L sucrose. For suspension culture, BY-2 cells were grown in 250 ml shake flasks containing 80 ml liquid medium and rotated at 95 rpm under continuous illumination at room temperature (22–24 °C). Subcultures were carried out every week with a 5% (v/v) inoculum density. Cultured cells were harvested by vacuum filtration. The cells were then dried in an oven at 70 °C for 48 h and weighed to determine the dry weight (DW). Cell biomass yield was expressed as grams of cell dry weight per liter medium (gDW/L).

### Protein extraction and Western blotting analysis

The culture media were directly used for Western blot assay. Intracellular proteins were extracted by grinding ~ 0.5 g cells (fresh weight) in liquid nitrogen, and then supplemented with SDS (sodium dodecyl sulfate) extraction buffer^10^ at a ratio of 1:2 (w/v). Samples were centrifuged at 13,000×*g* at 4 ℃ for 15 min and the supernatants were collected. For Western blot assay, protein samples were separated on a 4–20% precast Tris–HCl gel (Bio-Rad, Hercules, CA), and then electro-blotted onto a 0.2 μm nitrocellulose membrane (Bio-Rad, Hercules, CA). Protein blots were blocked with 3% (w/v) BSA in Tris-buffered saline (pH 7.5) containing 0.1% Tween^®^ 20. Recombinant EGFP and SCF products were detected using a rabbit anti-EGFP polyclonal antibody (ThermoFisher Scientific, Waltham, MA) and a rabbit-anti-SCF polyclonal antibody (ThermoFisher Scientific) as the primary antibody, respectively, and a goat-anti rabbit IgG (H + L)-peroxidase conjugated (Jackson Immuno Research labs, West Grove, PA) as the secondary antibody. Protein blots were then detected using the SuperSignal^®^ West Pico Chemiluminescence Substrate (ThermoFisher Scientific Inc., Waltham, MA). The blot images were captured either by the Li-Cor Odyssey Fc imaging system (Li-Cor Biosciences, NE) or exposed to an X-ray film.

### Laser scanning confocal imaging

Confocal imaging of the BY-2 cells grown in SH or MS medium was performed using a Nikon D-Eclipse C1 laser-scanning confocal head mounted on a Nikon Eclipse E800 microscope with either a 40× /0.8 W Nikon Fluor water immersion objective or a 100×/0.8 W Nikon Fluor oil immersion objective. The fluorescence of cells was detected at the following wavelengths: 488 nm excitation with a 525/50 nm filter for EGFP fluorescence and 543 nm excitation with a 595/50 nm filter for RFP fluorescence.

### Brefeldin A (BFA) treatment of BY-2 cells

BY-2 cells expressing (SP)_32_-EGFP and EGFP were cultured in SH medium for 8 days to reach mid‐exponential growth phase. The cultures were then supplemented with BFA to a final concentration of 10 µg/ml as described early^56^, and incubated at room temperature for 1, 2 and 4 h before the cells were examined by confocal microscopy and Western blotting assay.

### Total RNA extraction

Three biological replicates of BY-2 cells (wild-type or (SP)_32_-EGFP line) grown in SH and MS media were collected after 8 days of subculture. Total RNA was purified using the PureLink™ RNA Mini Kit (Invitrogen, Carlsbad, CA) following the manufacturer’s protocol. Concentration of the RNA was assessed NanoDrop™ OneC Microvolume UV–Vis Spectrophotometer (Thermo Fisher Scientific, Waltham, MA). RNA integrity was assessed using the RNA Nano 6000 Assay Kit of the Bioanalyzer 2100 system (Agilent Technologies, Santa Clara, CA).

### RT-qPCR procedure

One-step RT-qPCR was performed using SuperScript™ III Platinum™ One-Step qRT-PCR Kit (Invotrogen, Carlsbad, CA) in Bio-Rad CFX384 instrument (Hercules, CA, USA). Normalization was performed with 3 housekeeping genes, including *β*-*Actin*, *Elongation factor 1α* (*EF-1α*) and *L25 ribosomal protein* (*L25*) as reported earlier^57^. The expression level of (*SP*)_*32*_-*EGFP* and (*SP*)_*32*_*-SCF* gene, and four other endogenous genes, *alkane hydroxylase MAH1-like* (LOC107798865), *laccase-15-like* (LOC107829253), *polygalacturonase* (LOC107824442), and *vegetative cell wall protein gp1-like* (LOC107802007) were assessed. All the primers used for RT-qPCR are listed in Supplementary Table S7. Quality and data analysis were performed in Bio-Rad CFX Maestro Software using the 2^(−ΔΔCt)^ method, and the results for each gene were expressed as 2^(−ΔΔCt)^ values relative to the mean value of the control group.

### cDNA library construction and sequencing

The samples with an RNA integrity number (RIN) ≥ 7 were proceeded for cDNA library construction, sequencing, and data analysis by Novogene Corporation Inc. (Tianjin, China). Approximately 1.0 µg RNA per sample was used as input material for sequencing library preparation by using NEBNext^®^ Ultra™ RNA Library Prep Kit for Illumina^®^ (New England Biolabs, Ipswich, MA). Briefly, poly (A)^+^ mRNA was isolated from the total RNA sample using Oligo (dT) magnetic beads, and further fragmented with divalent cations. The first-strand cDNA was synthesized by using a random hexamer primer and M-MuLV reverse transcriptase, followed by the synthesis of the second-strand cDNA using DNA Polymerase I. Clusters of index-coded samples was generated on a cBot Cluster Generation System using PE Cluster Kit cBot-HS (Illumina, San Diego, CA). After clustering, the library preparations were sequenced on a HiSeq2500 Illumina platform and paired-end reads were generated. The sequencing data have been uploaded to NCBI Sequence Read Archive (Accession#: PRJNA931284).

### Bioinformatics analysis of raw RNA-Seq data

Quality control of raw reads of RNA-Seq in FASTQ format was conducted by fastp tool. The clean reads were mapped to the *Nicotiana tabacum* reference genome (Ntab-TN90, GenBank accession number: SAMN02316627) using HISAT2 software. Gene expression abundance was quantified by using FeatureCounts. Differential expression between the cells cultured in SH medium and MS medium (control) was analyzed using DESeq2 R package. Genes with an adjusted *P*-value < 0.05 found by DESeq2 were assigned as differentially expressed genes (DEGs), which were subjected to functional enrichment analysis. Gene Ontology (GO) enrichment analysis of DEGs was implemented by the ClusterProfiler R package to classify genes into three categories: molecular functions, cellular components, and biological processes. GO terms with corrected *P*-value < 0.05 were considered significantly enriched by DEGs. In addition, the ClusterProfiler R package was used for the Kyoto Encyclopedia of Genes and Genomes (KEGG) pathway analysis to identify enrichment pathways with the same cutoff value as the GO enrichment analysis.

### Statistical analysis

Assays of biomass and secreted protein yields were carried out with three replicates, and data are presented as the mean with accompanying standard deviation (*SD*). One-way analysis of variance (ANOVA) followed by a Tukey post hoc range test was used to determine differences among treatments with *p* < 0.05 considered to be significant.

### Supplementary Information

Supplementary Information.

## Data Availability

The RNA sequencing data generated during the current study are available in the NCBI Sequence Read Archive (Accession#: PRJNA931284). All the other datasets are available from the corresponding author on reasonable request.
