# Supplementary material for: Intracellular trafficking and glycosylation of hydroxyproline-O-glycosylation module in tobacco BY-2 cells is dependent on medium composition and transcriptome analysis
Source: Sci Rep. 2023 Aug 19;13:13506. doi: 10.1038/s41598-023-40723-3 (PMC10439957; doi:10.1038/s41598-023-40723-3)
Supplement: Supplementary file 1 — Supplementary Information. [file 41598_2023_40723_MOESM1_ESM.pdf]

Intracellular trafficking and glycosylation of hydroxyproline-*O*-glycosylation module in tobacco BY-2 cells is dependent on medium composition and transcriptome analysis

Uddhab Karki[1,2], Paula PerezSanchez[3], Sankalpa Chakraborty[1,2], Berry Dickey[3], Jacqueline Vargas[3], Ningning Zhang[1,2], Jianfeng Xu[1,2,4,*]

[1]Arkansas Biosciences Institute, Arkansas State University, Jonesboro, AR 72401

[2]Molecular BioSciences Program, Arkansas State University, Jonesboro, AR 72401

[3]Department of Biological Sciences, Arkansas State University, Jonesboro, AR 72401

[4]College of Agriculture, Arkansas State University, Jonesboro, AR 72401

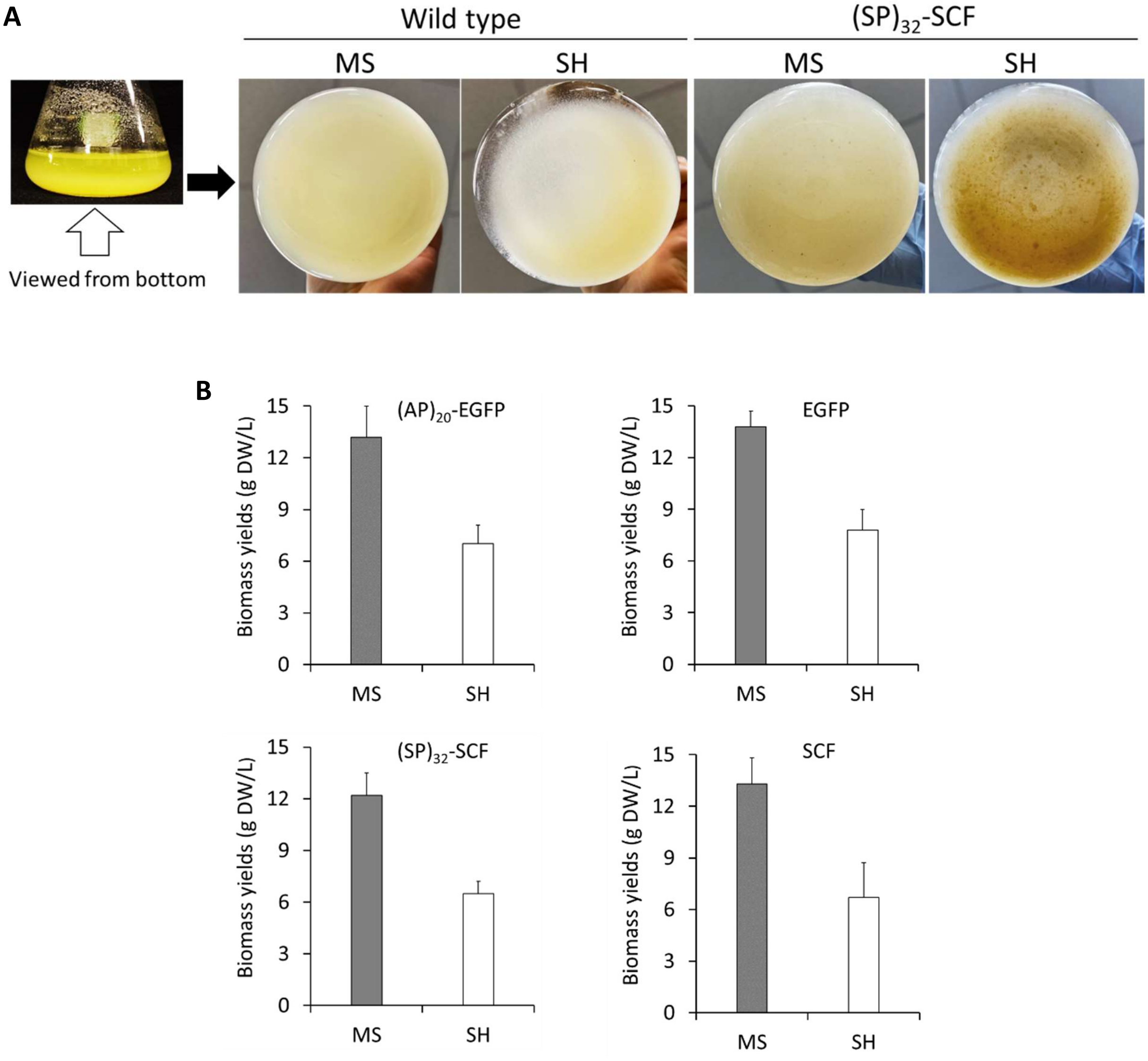

Supplementary Fig. S1 BY-2 cells cultured in liquid MS and SH medium. **(A)** Suspension culture of BY-2 cells viewed from the bottom of a flask; **(B)** Biomass yields of the cell culture in two types of medium. Error bars represent the standard deviation of three replicates (three cell cultures).

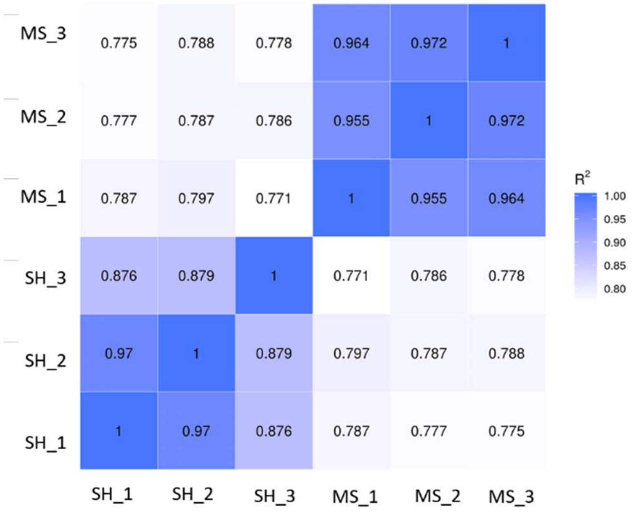

Supplementary Fig. S2 Heatmap of Pearson correlation between the samples, R^2 is coefficient of determination

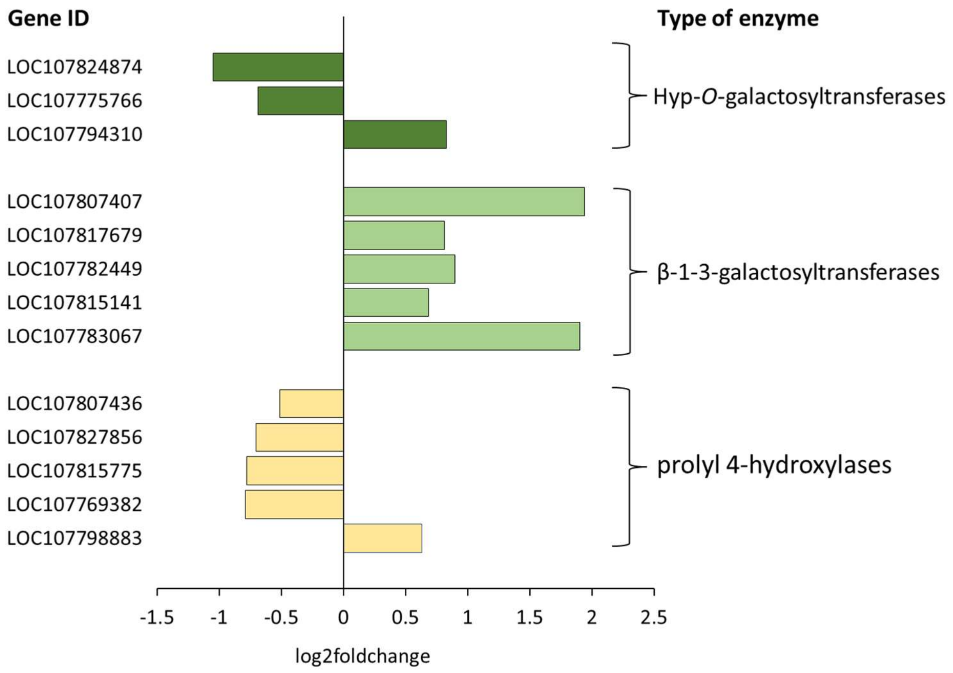

Supplementary Fig. S3 DEGs for prolyl 4-hydroxylase, Hyp-*O*-galactosyltransferase and β-1,3-galactosyltransferase identified in BY-2 cells cultured in SH medium vs. MS medium.

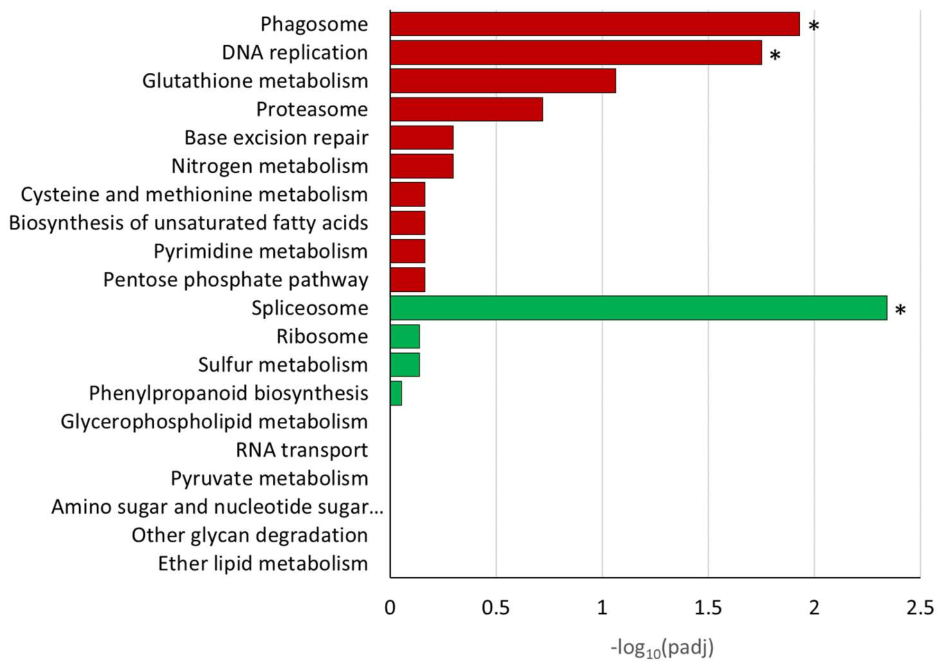

Supplementary Fig. S4 Kyoto Encyclopedia of Genes and Genomes (KEGG) pathway enrichment of DEGs. The top 10 top- and down- regulated cellular pathways in SH medium with respect to MS medium (control) are presented in red and green, respectively. * indicates significant difference at $p< 0.05$.

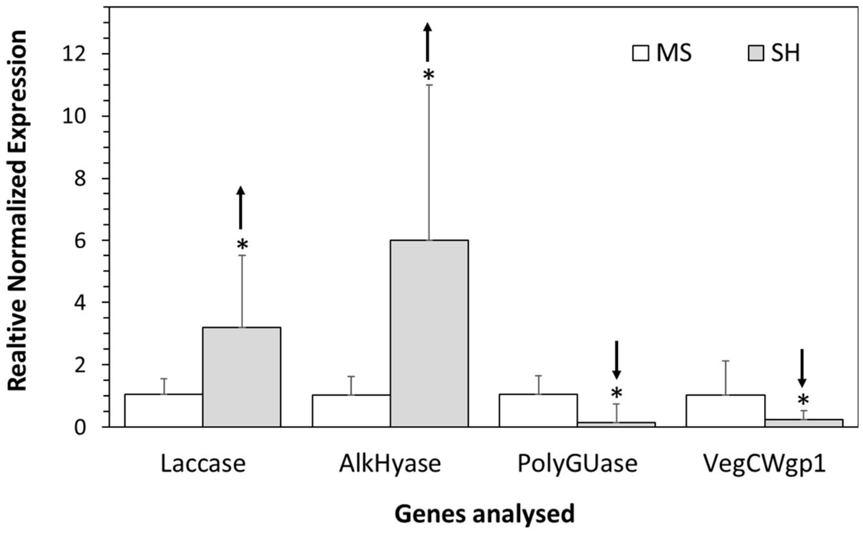

Supplementary Fig. S5 Validation of the RNA-Seq results of BY-2 cells cultured in SH medium vs. MS medium by RT-qPCR. Three biological replicates of BY-2 cells grown in SH and MS medium were collected after 8 days of subculture. The expression of two highly up-regulated genes, *alkane hydroxylase MAH1-like (AlkHyase)* and *laccase-15-like* (*Laccase*), and two highly down-regulated genes, probable *polygalacturonase* (*PolyGUase*) and *vegetative cell wall protein gp1-like* (*VegCWgp1*) were measured. Error bar represents the standard deviation of three biological replicates; *indicates significant difference relative to the sample from MS medium at $p < 0.05$. indicates upregulation, and downregulation.

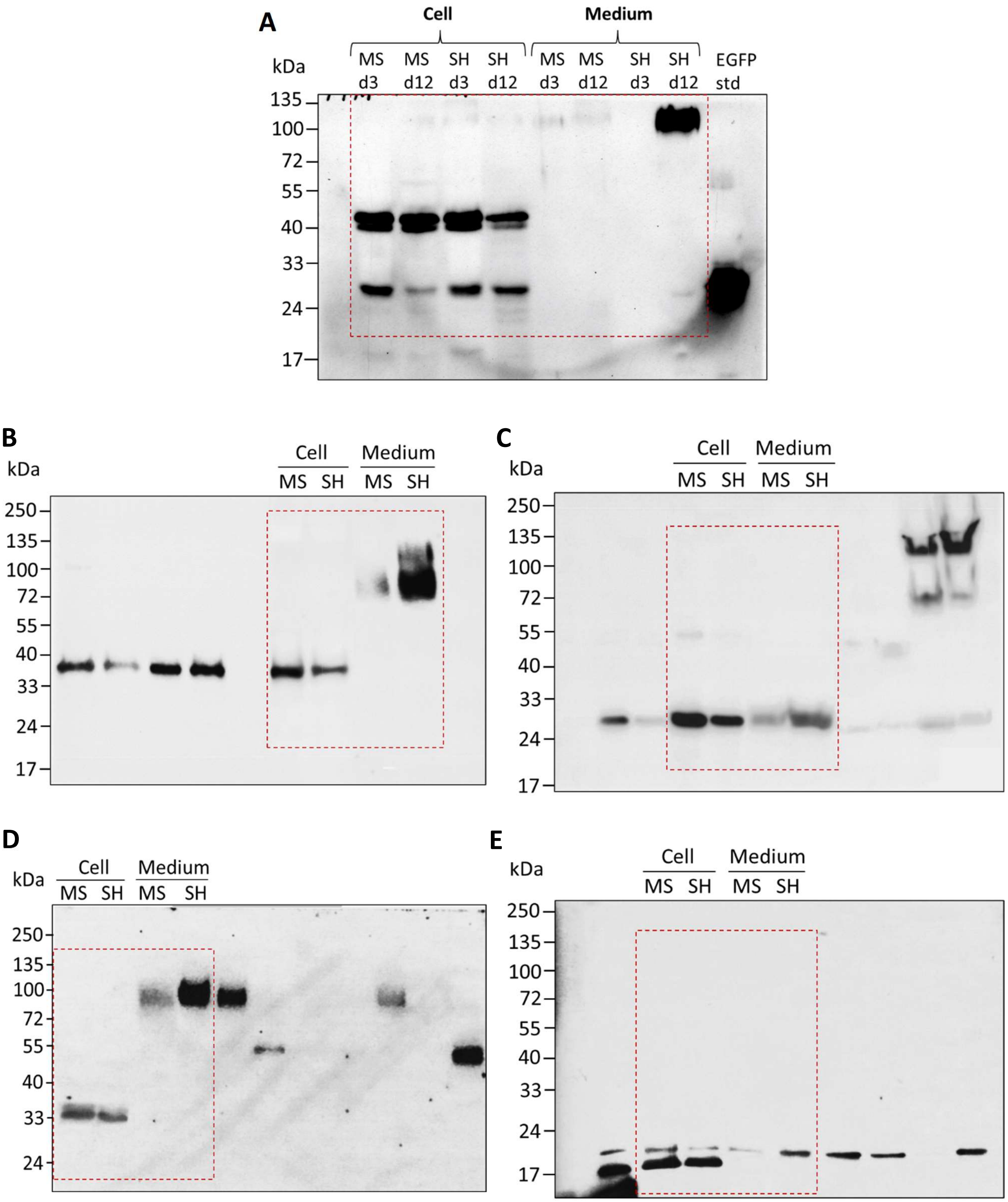

Supplementary Fig. S6 Original images of western blotting analysis. Panel A is related to Fig. 1. Area enclosed by red dash lines is used in Fig. 1C. Panel B, C, D, E are related to Fig. 2. Area enclosed by red dash lines are used in Fig. 2A, 2B, 2C, and 2D, respectively.

Supplementary Table S1. Relative recombinant protein yields of BY-2 cells cultured in MS and SH medium. The BY-2 cells and culture media were harvested for the assay after 12 days of culture. Sixteen µl protein extraction solution or 10 µl medium for each sample were loaded onto a SDS-PAGE gel. The relative protein yields, expressed as the ratio of protein produced in SH medium to protein produced in MS medium (SH/MS), were estimated based on densitometry with the Li-Cor's Image Studio™ Software. The Li-Cor imaging system provides a broad, linear and dynamic range to accurately quantify both strong and weak bands. Data are presented as the mean of three parallel sample assays with standard deviation.

**Recombinant proteins**	$(SP)_{32}$-EGFP	$(AP)_{20}$-EGFP	EGFP	$(SP)_{32}$-SCF	SCF
**Intracellular protein (SH/MS)[a]**	0.70±0.04	0.68±0.03	0.54±0.05	0.81±0.05	0.92±0.04
**Secreted protein (SH/MS)[b]**	150.2±10.3	21.4±3.2	1.8±0.2	10.3±1.9	2.2±0.1

[a]: ratio of intracellular protein in SH medium to MS medium; [b]: ratio of secreted protein in SH medium to MS medium

Supplementary Table S2. Degree of glycosylation of HypGP-tagged proteins produced by BY-2 cells cultured in MS and SH medium. The BY-2 cell cultures were harvested for the assay after 12 days of culture. The degree of glycosylation (Hyp-*O*-glycosylation) was estimated by calculating the percentage of glycosylated protein accounting for the total proteins produced by culture cells, including both intracellular and secreted proteins. Data are presented as the mean of three parallel sample assays with standard deviation.

**HypGP-tagged proteins**	$(SP)_{32}$-EGFP	$(AP)_{20}$-EGFP	$(SP)_{32}$-SCF
**MS**	5.3±1.4	31.2±2.3	26.5±2.7
**SH**	96.2±0.8	92.5±1.2	72.6±2.1

Supplementary Table S3. Compositions of SH and MS medium

Medium components (mg/L)	SH	MS
Macronutrients		
NH_4NO_3		1650
KNO_3	2500	1900
$CaCl_2.2H_2O$	151	440
$MgSO_4.7H_2O$	195.4	370
KH_2PO_4		170
$NH_4H_2PO_4$	300	
$(NH_4)_2SO_4$		
$NaH_2PO_4.H_2O$		
Micronutrients		
KI	1	0.83
H_3BO_3	5	6.2
$MnSO_4.H_2O$	10	22.3
$ZnSO_4.7H_2O$	1	8.6
$Na_2MoO_4.2H_2O$	0.1	0.25
$CuSO_4.5H_2O$	0.2	0.025
$CoCl_2.6H_2O$	0.1	0.025
Na_2EDTA	20	37.3
$FeSO_4.7H_2O$	15	27.8
Vitamins		
Inositol	1000	100
Glycine		2
Thiamine HCl	5	0.1
Pyridoxine HCl	0.5	0.5
Nicotinic acid	5	0.5

Supplementary Table S4. Mapping of total reads obtained from tobacco BY-2 cells with *N. tabacum* reference genome

Sample Name	Total reads	Total mapped reads	Total mapping rate	Uniquely mapped reads	Uniquely mapping rate
SH_1	44256940	42777998	96.66%	41440934	93.64%
SH_2	39654788	41714833	96.76%	40403614	93.83%
SH_3	43159750	38370253	96.65%	37206499	93.61%
MS_1	40553864	38849644	95.80%	37675628	92.90%
MS_2	40996868	39278257	95.81%	38054964	92.82%
MS_3	42137936	40372752	95.81%	39186109	92.99%

Supplementary Table S5. **List of consistently significant highly downregulated genes in BY-2 cells cultured in SH medium vs. MS medium**

Gene Name	log_2foldchange	NCBI gene Annotation	p-adjusted value
LOC107824442	-9.095	probable polygalacturonase	3.203E-97
LOC107801990	-8.515	aspartyl protease AED3-like	5.043E-126
LOC107761934	-8.512	GDSL esterase/lipase LIP-4-like	1.787E-58
LOC107802007	-7.989	vegetative cell wall protein gp1-like	3.554E-235
LOC107794477	-7.429	osmotin-like protein	7.775E-163
LOC107823460	-7.159	zinc transporter 11-like	2.586E-123
LOC107801270	-7.085	inorganic pyrophosphatase 1-like	3.643E-85
LOC107830562	-6.975	uncharacterized LOC107830562	9.076E-59
LOC107806163	-6.948	aspartyl protease AED3-like	3.649E-68
LOC107799202	-6.946	basic form of pathogenesis-related protein 1	1.110E-91
LOC107783342	-6.874	purple acid phosphatase 2-like	1.692E-76
LOC107817132	-6.457	inorganic pyrophosphatase 1-like	1.292E-71
LOC107763440	-6.194	aspartyl protease family protein At5g10770-like	2.592E-76
LOC107780309	-6.163	BURP domain-containing protein 5-like	1.270E-73
LOC107767515	-5.925	triacylglycerol lipase 2-like	2.625E-66
LOC107807054	-5.641	36.4 kDa proline-rich protein-like	4.482E-119
LOC107818543	-5.629	zinc transporter 11-like	7.397E-119
LOC107765474	-5.585	cytochrome c biogenesis protein CCS1%2C chloroplastic-like	7.888E-54
LOC107816752	-5.519	beta-xylosidase/alpha-L-arabinofuranosidase 2-like	4.976E-173
LOC107801151	-5.480	glucan endo-1%2C3-beta-glucosidase%2C basic vacuolar	8.760E-70
LOC107832840	-5.287	glycine-rich protein 2	9.321E-80
LOC107810524	-5.281	LRR receptor-like serine/threonine-protein kinase FLS2	4.121E-74
LOC107813878	-5.274	36.4 kDa proline-rich protein-like	6.462E-153
LOC107767187	-5.258	proline-rich receptor-like protein kinase	2.790E-129
LOC107830556	-5.234	copper transporter 1-like	1.261E-75
LOC107803188	-5.207	snakin-2	4.426E-54
LOC107805330	-4.807	indole-3-acetic acid-induced protein ARG7-like	1.992E-52
LOC107828040	-4.805	uncharacterized LOC107828040%2C transcript variant X2	3.106E-74
LOC107823411	-4.216	glucan endo-1%2C3-beta-glucosidase%2C basic vacuolar	1.165E-79
LOC107805188	-4.107	pyridoxal 5'-phosphate synthase subunit PDX1	1.297E-60
LOC107793330	-3.994	glycine-rich domain-containing protein 1-like	3.091E-109
LOC107810914	-3.961	uncharacterized LOC107810914	3.993E-68
LOC107824608	-3.807	lipoxygenase homology domain-containing protein 1-like	1.458E-67
LOC107809983	-3.789	glycine-rich domain-containing protein 1-like	2.390E-89
LOC107794478	-3.381	osmotin-like protein	1.370E-66
LOC107821516	-3.310	receptor-like protein kinase HAIKU2	5.902E-54
LOC107770491	-3.305	EIN3-binding F-box protein 1-like	5.572E-56

Supplementary Table S6. List of consistently significant highly upregulated genes in BY-2 cells cultured in SH media

Gene Name	log_2(foldchange)	NCBI gene Annotation	p-adjusted value
LOC107798865	9.820	alkane hydroxylase MAH1-like	1.265E-67
LOC107796758	6.662	transcription factor ORG2-like%2C	4.884E-44
LOC107767650	6.469	auxin-responsive protein IAA9-like	4.109E-50
LOC107824061	6.108	dirigent protein 23-like	1.769E-52
LOC107808555	5.979	D-3-phosphoglycerate dehydrogenase	5.786E-40
LOC107807137	5.585	ervatamin-B-like	2.712E-46
LOC107805563	5.564	uclacyanin-3-like	1.831E-47
LOC107794678	5.323	suberization-associated anionic peroxidase 1-like	2.555E-42
LOC107814800	5.310	non-specific lipid-transfer protein 2	2.690E-42
LOC107785583	4.949	uncharacterized LOC107785583	3.375E-41
LOC107819206	4.679	UPF0047 protein YjbQ-like	2.163E-39
LOC107816672	4.579	uncharacterized LOC107816672	7.253E-80
LOC107811292	4.477	polyphenol oxidase E%2C	2.502E-54
LOC107767528	4.389	superoxide dismutase [Cu-Zn]%2C	2.425E-50
LOC107806960	4.288	superoxide dismutase [Cu-Zn]%2C chloroplastic	6.599E-41
LOC107784914	4.221	cytochrome P450 78A7-like	1.583E-42
LOC107829253	4.175	laccase-15-like	3.883E-40
LOC107793829	4.171	uncharacterized LOC107793829	9.133E-52
LOC107802807	4.164	probable pectate lyase 8	2.397E-37
LOC107766787	4.092	transcription factor bHLH63-like	6.733E-35
LOC107803559	3.993	homeobox-leucine zipper protein HOX3-like	4.126E-53
LOC107830193	3.894	inactive protein kinase SELMODRAFT_444075-like	6.013E-34
LOC107824504	3.839	deoxyuridine 5'-triphosphate nucleotidohydrolase	1.809E-34
LOC107787575	3.412	uncharacterized LOC107787575	4.195E-66
LOC107810930	3.391	uncharacterized LOC107810930	2.093E-36
LOC107788814	3.357	bifunctional L-3-cyanoalanine synthase	2.807E-55
LOC107800430	3.192	origin of replication complex subunit 6-like	2.308E-36
LOC107824959	3.051	auxin-induced protein 22D-like	1.743E-65
LOC107775979	2.433	uncharacterized LOC107775979	1.978E-35
LOC107806775	2.413	translocon-associated protein subunit beta-like	9.172E-36
LOC107760028	2.398	transcription factor bHLH122-like%2C	8.300E-42
LOC107782081	2.280	homeobox-leucine zipper protein MERISTEM L	1.257E-35
LOC107814855	2.172	tubulin beta-1 chain	1.224E-35
LOC107820374	2.110	auxin-repressed 12.5 kDa protein-like	8.546E-45
LOC107818269	2.003	tubulin beta-2 chain-like	8.885E-46

Supplementary Table S7. Listed of primers used for RT-qPCR analysis

Primer name	Primer sequence	Gene analyzed
EGFP-F	5'- gcacaagctggagtacaacta -3'	*$(SP)_{32}$-EGFP*
EGFP-R	5'- tgttgtggcggatcttgaa -3'	
SCF-F	5'- ataaccctcaaatatgtcccc -3'	*$(SP)_{32}$-SCF*
SCF-R	5'- gtccagaagatcagtcaagc -3'	
AlkHyase-F	5'- tgtttgacattatgggagatgga -3'	*alkane hydroxylase MAH1-like gene (LOC107798865)*
AlkHyase-R	5'- ggaacttgggatggctcatta -3'	
Laccase-F	5'- cccaagcctcatgctgaaata -3'	*laccase-15-like gene (LOC107829253)*
Laccase-R	5'- gacctcctgaggcagtaaattc -3'	
PolyGUase-F	5'- agaatcccattgccatctcag -3'	*probable polygalacturonase gene (LOC107824442)*
PolyGUase-R	5'- tcttccctctgctcctactaaa -3'	
VegCWgp1-F	5'- ccttctcctccaccttcaataat -3'	*vegetative cell wall protein gp1-like gene (LOC107802007)*
VegCWgp1-R	5'- caggtatgagtggtgggataag -3'	
Actin-F	5'- cctgaggtccttttccaacca -3'	*Actin (Tac9) gene (X69885)*
Actin-R	5'- ggattccggcagcttccatt -3'	
EF-1α-F	5'- tgagatgcaccacgaagctc -3'	*Elongation factor 1a (EF-1a) gene (AF120093)*
EF-1α-R	5'- ccaacattgtcaccaggaagtg -3'	
L25-F	5'- cccctcaccacagagtctgc -3'	*L25 ribosomal protein gene (L18908)*
L25-R	5'- aagggtgttgttgtcctcaatctt -3'	
